# METTL14 promotes lipid metabolism reprogramming and sustains nasopharyngeal carcinoma progression via enhancing m^6^A modification of ANKRD22 mRNA

**DOI:** 10.1002/ctm2.1766

**Published:** 2024-07-17

**Authors:** Lvyuan Li, Qiling Tang, Junshang Ge, Dan Wang, Yongzhen Mo, Yijie Zhang, Yumin Wang, Fang Xiong, Qijia Yan, Qianjin Liao, Can Guo, Fuyan Wang, Ming Zhou, Bo Xiang, Zhaoyang Zeng, Lei Shi, Pan Chen, Wei Xiong

**Affiliations:** ^1^ NHC Key Laboratory of Carcinogenesis and Hunan Key Laboratory of Cancer Metabolism Hunan Cancer Hospital and the Affiliated Cancer Hospital of Xiangya School of Medicine, Central South University Changsha China; ^2^ Key Laboratory of Carcinogenesis and Cancer Invasion of the Chinese Ministry of Education Cancer Research Institute and School of Basic Medicine Sciences, Central South University Changsha China; ^3^ Department of Otolaryngology Head and Neck Surgery Xiangya Hospital, Central South University Changsha China; ^4^ Department of Pathology the Second Xiangya Hospital, Central South University Changsha China

**Keywords:** ANKRD22, lipid metabolism reprogramming, m^6^A, METTL14, nasopharyngeal carcinoma

## Abstract

**Background:**

N^6^‐methyladenosine (m^6^A) modification is essential for modulating RNA processing as well as expression, particularly in the context of malignant tumour progression. However, the exploration of m^6^A modification in nasopharyngeal carcinoma (NPC) remains very limited.

**Methods:**

RNA m^6^A levels were analysed in NPC using m^6^A dot blot assay. The expression level of methyltransferase‐like 14 (METTL14) within NPC tissues was analysed from public databases as well as RT‐qPCR and immunohistochemistry. The influences on METTL14 expression on NPC proliferation and metastasis were explored via in vitro as well as in vivo functional assays. Targeted genes of METTL14 were screened using the m^6^A and gene expression profiling microarray data. Actinomycin D treatment and polysome analysis were used to detect the half‐life and translational efficiency of ANKRD22. Flow cytometry, immunofluorescence and immunoprecipitation were used to validate the role of ANKRD22 on lipid metabolism in NPC cells. ChIP‐qPCR analysis of H3K27AC signalling near the promoters of METTL14, GINS3, POLE2, PLEK2 and FERMT1 genes.

**Results:**

We revealed METTL14, in NPC, correlating with poor patient prognosis. In vitro and in vivo assays indicated METTL14 actively promoted NPC cells proliferation and metastasis. METTL14 catalysed m^6^A modification on ANKRD22 messenger ribonucleic acid (mRNA), recognized by the reader IGF2BP2, leading to increased mRNA stability and higher translational efficiency. Moreover, ANKRD22, a metabolism‐related protein on mitochondria, interacted with SLC25A1 to enhance citrate transport, elevating intracellular acetyl‐CoA content. This dual impact of ANKRD22 promoted lipid metabolism reprogramming and cellular lipid synthesis while upregulating the expression of genes associated with the cell cycle (GINS3 and POLE2) and the cytoskeleton (PLEK2 and FERMT1) through heightened epigenetic histone acetylation levels in the nucleus. Intriguingly, our findings highlighted elevated ANKRD22‐mediated histone H3 lysine 27 acetylation (H3K27AC) signals near the METTL14 promoter, which contributes to a positive feedback loop perpetuating malignant progression in NPC.

**Conclusions:**

The identified METTL14‐ANKRD22‐SLC25A1 axis emerges as a promising therapeutic target for NPC, and also these molecules may serve as novel diagnostic biomarkers.

## Graphical abstract

- METTL14 promotes the proliferation, invasion and migration of nasopharyngeal carcinoma cells.
- METTL14 enhances ANKRD22 mRNA stability and translation through the m^6^A‐IGF2BP2 dependent pathway.
- ANKRD22 binds to SLC25A1 to promote lipid metabolism reprogramming, leading to the upregulation of proliferation, invasion and migration‐related gene expression via enhancing H3K27AC, which in turn activates METTL14 transcription.

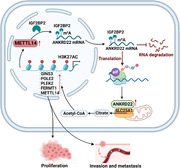

## 1 BACKGROUND

Nasopharyngeal carcinoma (NPC) originates from the nasopharyngeal mucosa and is a malignant tumour predominantly prevalent in East and Southeast Asia.^1^ The aetiology of NPC involves elements like Epstein‐Barr virus (EBV) infection,^2^ genetic background,^3^, ^4^ environment influences^5^ and dietary habits.^6^ Due to its inconspicuous onset, subtle early symptoms, and pronounced invasive and proliferative characteristics,^7^ most patients present with lymph node metastasis when diagnosed.^8^ While radiotherapy stands as the primary clinical intervention for NPC,^9^ the principal challenges lie in recurrence and metastasis, constituting the major causes of treatment failure.^10^ The pathogenesis of NPC encompasses numerous abnormalities in gene expression, and the underlying molecular mechanisms remain incompletely elucidated. Recent studies have uncovered the significance of dysregulated N^6^‐methyladenosine (m^6^A) modification on messenger ribonucleic acid (mRNA) in governing gene expression through diverse mechanisms, playing a crucial role in the development of malignant tumours.^11^, ^12^, ^13^ Nevertheless, our current understanding of m^6^A modification in the pathogenesis of NPC is quite limited.

m^6^A modification involves the methylation of the sixth nitrogen of adenosine. This modification is dynamically regulated primarily with m^6^A writers and erasers.^14^, ^15^, ^16^, ^17^, ^18^ Subsequently, m^6^A‐modified mRNA is recognized by various specific m^6^A recognition proteins (readers), which determine the fate of mRNA in terms of splicing and processing,^19^, ^20^ RNA stability^21^, ^22^, ^23^ and translation efficiency.^23^, ^24^ The key writers of m^6^A modification include methyltransferase‐like 14 (METTL14), Wilms tumour 1‐associated protein (WTAP) and methyltransferase‐like 3 (METTL3). It has been shown that WTAP‐mediated m^6^A modification of lncRNA DIAPH1‐AS1 in NPC increases its stability, thereby promoting the growth and metastasis of NPC.^25^ Additionally, WTAP‐mediated m^6^A methylation on TEA domain transcription factor 4 has been linked to enhanced stability, contributing to NPC metastasis and chemotherapy resistance.^26^ METTL3 has been identified in mediating m^6^A modification of the lncRNA SUCLG2‐AS1, subsequently promoting metastasis and radiotherapy resistance in NPC.^27^ Through the analysis of NPC Gene Expression Omnibus (GEO) datasets GSE12452 and GSE61218, we observed a significant upregulation of METTL14 in NPC. It is yet unknown, however, what METTL14's biological roles and possible regulation mechanisms might be in NPC.

We verified in our investigation that METTL14 expression was indeed elevated in clinical NPC samples, correlating with poor prognosis in patients. Our work demonstrated the pivotal effect of METTL14 in promoting NPC cells growth and metastasis through both in vivo and in vitro assays. Subsequent analysis of NPC m^6^A and gene expression profiling microarray data,^28^ coupled with functional validation, pinpointed ankyrin repeat domain 22 (ANKRD22) as a key downstream target regulated by METTL14. ANKRD22 was found to interact with the citrate transporter protein solute carrier family 25 member 1 (SLC25A1), fostering the production of acetyl coenzyme A (acetyl‐CoA). This, in turn, facilitated lipid metabolic reprogramming and epigenetic modifications. On one hand, ANKRD22 enhanced histone H3 lysine 27 acetylation (H3K27AC) near the promoters of genes related to the cell cycle and cytoskeleton, including GINS Complex Subunit 3 (GINS3), DNA Polymerase Epsilon 2 (POLE2), Pleckstrin 2 (PLEK2) and FERM Domain Containing Kindlin 1 (FERMT1), thereby promoting proliferation and metastasis of NPC. Simultaneously, ANKRD22 increased H3K27AC near the METTL14 promoter, resulting in the upregulation of METTL14 expression. This intricate regulatory mechanism constituted a positive feedback loop within the METTL14‐m^6^A‐ANKRD22 axis.

## 2 METHODS

### 2.1 NPC clinical samples

The clinical samples in this study encompassed various tissue types, including fresh NPC tissues, control tissues from chronic nasopharyngeal inflammation and paraffin‐embedded NPC tissues. For RNA extraction and RT‐qPCR, we utilized 7 chronic nasopharyngeal inflammation tissues and 28 fresh NPC tissues obtained from Xiangya Hospital of Central South University (Table S1). The paraffin‐embedded tissue sections, comprising 80 NPC tissues and 42 adjacent non‐tumour nasopharyngeal epithelial (NPE) tissues with clinical prognostic information (Table S2), were sourced from the Affiliated Cancer Hospital of Xiangya School of Medicine, Central South University.

These sections were employed for immunohistochemistry (IHC) to study the association between METTL14 as well as ANKRD22 expression and patient prognosis. Furthermore, an additional set of paraffin‐embedded tissue sections, consisting of 70 NPC tissues and 40 adjacent non‐tumour NPE tissues (Table S3), obtained from Xiangya Hospital of Central South University, were used for IHC analysis to explore the correlation between the expression of METTL14, ANKRD22, GINS3, POLE2, PLEK2 and FERMT1. Histopathological examination verified the validity of all NPC clinical samples.

### 2.2 Cell culture, plasmids, small interfering RNAs and transfection

At the Institute of Cancer Research, Central South University, the NPC cell lines (CNE2, HONE1) were maintained. All NPC cells were grown on a moist incubator with 37°C and 5% CO_2_ using RPMI‐1640 medium (VivaCell) added with 10% foetal bovine serum (OriCell).

The METTL14‐Flag overexpression plasmid was purchased from Addgene (Addgene). The ANKRD22‐Flag overexpression plasmid, full‐length ANKRD22 plasmid, SLC25A1‐His overexpression plasmid and four truncated ANKRD22 plasmids were purchased from YouBio (YouBio). The pMIR‐Luciferase‐Reporter plasmids with insertion of ANKRD22 5′ UTR wild‐type and mutant sequences (mutation of adenosine (A) to cytosine (C) in two m^6^A motif at positions 130 and 140 nt) and three truncated SLC25A1 plasmids were purchased from Tsingke (Tsingke). The siRNAs targeting METTL14, ANKRD22, IGF2BP1/2/3 and YTHDF1/2/3 were bought from RiboBio (RiboBio). Table S4 lists the sequences of the siRNAs. Plasmids were transfected with Lipofectamine 3000 (Life Technologies), while siRNAs were transfected using Hiperfect (Qiagen). The SLC25A1 inhibitor CTPI‐2 (MedChemExpress) was diluted to a concentration of 50 µM for cell treatment. The histone acetyltransferase inhibitor C646 (Selleck) was diluted to a concentration of 20 µM for cell treatment.

### 2.3 RT‐qPCR

The HiScript cDNA Synthesis Kit (Novoprotein) was used to reverse transcribe RNA into cDNA. For RT‐qPCR analysis, SYBR Green (Bimake) was utilized. β‐actin served as the internal reference. Table S5 contains a list of primer sequences that were employed.

### 2.4 Western blotting

Extraction of cells’ proteins was performed with RIPA buffer (Yamei) containing a protease inhibitor cocktail (TargetMol). Protein solutions were separated with SDS‐PAGE (8−12%) before being transferred to 0.2 µm polyvinylidene fluoride (PVDF) membranes. The membrane was then blotted using 10% skim milk at room temperature over 1 h before treating with primary antibody for 12 h at 4°C. Lastly, the membrane was treated at room temperature approximately 2 h with the appropriate secondary antibody applied. An ECL detection device (Millipore) was used to find protein bands. A loading control was applied using β‐tubulin. Table S6 shows the antibodies applied.

### 2.5 Immunohistochemistry

Paraffin‐embedded sections of mouse xenograft tissues and clinical NPC tissues were subjected to IHC using the UltraSensitive SP (Mouse/Rabbit) IHC kit (MXB). The staining was graded based on the percentage of stained cells and the staining intensity. Intensity was assessed as none (0), faint (1), moderate (2) and strong (3); and the percentage of stained cells was assessed as none (0), ≤25% (1), 25−50% (2), 50−75% (3) and > 75% (4). The intensity score plus the percentage score were multiplied to arrive at the final score. Two professional pathologists independently evaluated the stained sections. Table S6 shows the antibodies applied.

### 2.6 Immunofluorescence

For mitochondrial staining, medium containing 200 nM Mito‐Tracker Red CMXRos (Beyotime) was pre‐warmed to 37°C, and then NPC cells were perfused at 37°C for 30 min. The membranes were penetrated using 0.25% Triton X‐100 and subsequently closed off with 5% BSA after fixing with 4% paraformaldehyde. Incubate primary antibody at 4°C through the night, and subsequently incubate with the corresponding fluorescently labelled secondary antibody at 37°C for 1 h. DAPI (Beyotime) was used as a counterstain for the cell nuclei and pictures were acquired with a laser confocal microscope (Leica). Table S6 shows the antibodies applied.

### 2.7 Wound healing and transwell assays

Sterile 10 µL pipette tips were used to create uniform scratches on transfected cells in a 6‐well plate for the wound healing assay. Images were captured under a microscope at 0 and 24 h, respectively. For the transwell invasion and migration assay, cells suspended in RPMI‐1640 medium were incorporated into the upper chamber of a transwell insert with or without 10% Matrigel (BD), while RPMI‐1640 medium with 20% foetal bovine serum would be additionally incorporated onto the lower chamber. Cells were immobilized by 4% paraformaldehyde, subsequently, they were coloured by 0.1% crystal violet, and observed under an inverted phase‐contrast microscope.

### 2.8 MTT and colony formation assays

A 96‐well plate was used, with 1000 transfected cells put into each well for the MTT assay. Each well received 20 µL of MTT (Beyotime), and the mixture underwent incubation for 4 h at 37°C. After that, 200 µL of DMSO was added after the culture medium was withdrawn. At 490 nm, absorbance was measured with a microplate reader. Two thousand transfected cells were cultivated in each well of the 12‐well plate throughout 7 days in order to carry out colony formation assay. The cells were coloured by 0.1% crystal violet for analysis after fixing the cells with 4% paraformaldehyde.

### 2.9 RNA m^6^A dot blot

Briefly, the isolated total RNA was combined with saline sodium citrate (SSC) buffer after being denatured for 5 min at 65°C. The RNA samples were then transferred onto an Amersham Hybond‐N+ membrane (Solarbio). The membranes were UV crosslinked for 5 min, then stained with 0.02% methylene blue (Coolaber) and scanned for imaging as a loading control. Alternatively, the UV‐crosslinked membranes were blocked over 1 h using 5% skim milk and then treated with anti‐m^6^A antibody for an entire night at 4°C. Dot blot signals were visualized using an imaging system after incubation with the secondary antibody. Table S6 shows the antibodies applied.

### 2.10 Animal experiments

Four sets of six female BALB/C nude mice each were freely assigned. A total of 2×10^6^ CNE2 cells transfected with empty vector, negative control (NC), METTL14 overexpression vector or METTL14 siRNA, respectively, were injection subcutaneously or via tail vein. Tumour growth was observed every 5 days for the subcutaneous tumour model, and tumour size was evaluated by calculating the tumour volume V = 1/2 x (length) x (width) x (width). The tumour tissue was removed 30 days following the subcutaneous injection. The tumour masses were then weighed. Mice that received a tail vein injection of cells were slaughtered by cervical dislocation 60 days. Lung tissue was taken out, and each mouse's lung surface was counted for the number of metastatic nodules that indicated the extent of tumour metastasis.

### 2.11 Hematoxylin‐eosin staining

Mouse tissue slices immersed in paraffin were heated for 2 h at 65°C. The nuclei were coloured with hematoxylin solution (Coolaber) followed by the cytoplasm with eosin solution (Coolaber) following xylene dewaxer and gradient ethanol hydration. Finally, the sections were sealed and preserved with neutral resin.

### 2.12 Actinomycin D treatment

NPC cells were treated with Actinomycin D (Abmole) at 5 µg/mL, followed by RNA collection for RT‐qPCR at 0, 2 and 4 h. Table S5 contains a list of primer sequences that were employed.

### 2.13 Polysome analysis

The cells on dishes were treated for 5 min in a humidified incubator at 37°C with 5% CO_2_ with 100 µg/mL cycloheximide (Beyotime). Subsequently, cells were collected by gentle scraping and centrifugation after washing the cells twice with pre‐cooled PBS supplemented with 100 µg/mL cycloheximide. By adding hypotonic buffer supplemented with 100 µg/mL cycloheximide, 100 U RNase inhibitor, 0.5% sodium deoxycholate, 2 mM DTT and 0.5% Triton X‐100, the cells were resuspended. Following centrifugation, the supernatant was extracted. Ten percent of the supernatant, representing the input, was retained, while the remaining portion was transferred to tubes of an ultracentrifuge with a sucrose gradient (5%, 10%, 25%, 35% and 50%) that had been allowed to stand overnight. The mixture was centrifuged over 2 h at 4°C at 32,000 rpm. Subsequent to centrifugation, RNA from distinct ribosome fractions was extracted and subjected to analysis through RT‐qPCR. Table S5 contains a list of primer sequences that were employed.

### 2.14 MeRIP RT‐qPCR

Total RNA was extracted using TRIzol reagent (Accurate). Following the manufacturer's instructions, riboMeRIP m^6^A Transcriptome Profiling Kit (RiboBio) was then used. The RNA enriched by the anti‐m^6^A antibody was purified and recovered by Magen Hipure Serum/plasma miRNA Kit (Magen), and analysed by RT‐qPCR. Table S5 contains a list of primer sequences that were employed.

### 2.15 RNA pull‐down

Full‐length ANKRD22 mRNA, ANKRD22 5′ UTR‐wt and 5′ UTR‐mut were transcribed in vitro using the Biotin RNA Labeling Mix kit (Roche) and T7 RNA polymerase (Promega). The cell lysate was incubated with the biotin‐labelled RNA at room temperature for 2 h, followed by the addition of 50 µL Streptavidin Magnetic Beads (Invitrogen), and rotated and incubated at 4°C overnight. Finally, western blotting was used to examine the bound proteins.

### 2.16 Lipid staining

Cells were stained with BODIPY 493/503 (GLPBIO) for 15 min at 37°C protected from the light. Applying a laser confocal microscope (Leica), pictures were captured after the cells were counterstained with DAPI for 5 min. Alternatively, cells were collected for flow cytometry and data were analysed using FlowJo v10 software (Treestar).

### 2.17 Measurement of citrate and acetyl‐CoA

After 48 h of cell transfection, the levels of citrate and acetyl‐CoA were measured using the Mitochondrial Citrate Assay Kit (ZCIBIO) and the CheKine Acetyl‐CoA Assay Kit (Abbkine), respectively, following the manufacturer's instructions.

### 2.18 Immunoprecipitation

Antibodies were mixed with 50 µL of protein A/G magnetic beads (Bimake) and incubated at room temperature for 2 h with rotation, followed by the addition of cell lysates for overnight incubation at 4°C with rotation. The enriched proteins were analysed by western blotting. Table S6 shows the antibodies applied.

### 2.19 RNA immunoprecipitation and chromatin immunoprecipitation

The Magna RIP kit (Millipore) was used for RIP to analyse the interaction between METTL14, IGF2BP2 and ANKRD22 mRNA, followed by RT‐qPCR analysis. Additionally, following the manufacturer's instructions, the ChIP Assay Kit (Beyotime) was used for ChIP, followed by RT‐qPCR analysis. Table S5 contains a list of primer sequences that were employed.

### 2.20 Statistical analysis

Statistical analysis was carried out with GraphPad Prism 8.0 software. Student's *t*‐test was employed to compare differences between the two groups. One‐way ANOVA was used when comparing more than two groups. Overall survival curves were plotted using the Kaplan−Meier method and compared using the log‐rank test. All data are presented as mean ± standard deviation (SD). *p* < 0.05 was considered statistically significant. *p < 0.05; **p < 0.01; ***p < 0.001; ****p < 0.0001.

## 3 RESULTS

### 3.1 METTL14 is highly expressed in NPC and associated with poor prognosis of patients

**FIGURE 1:**
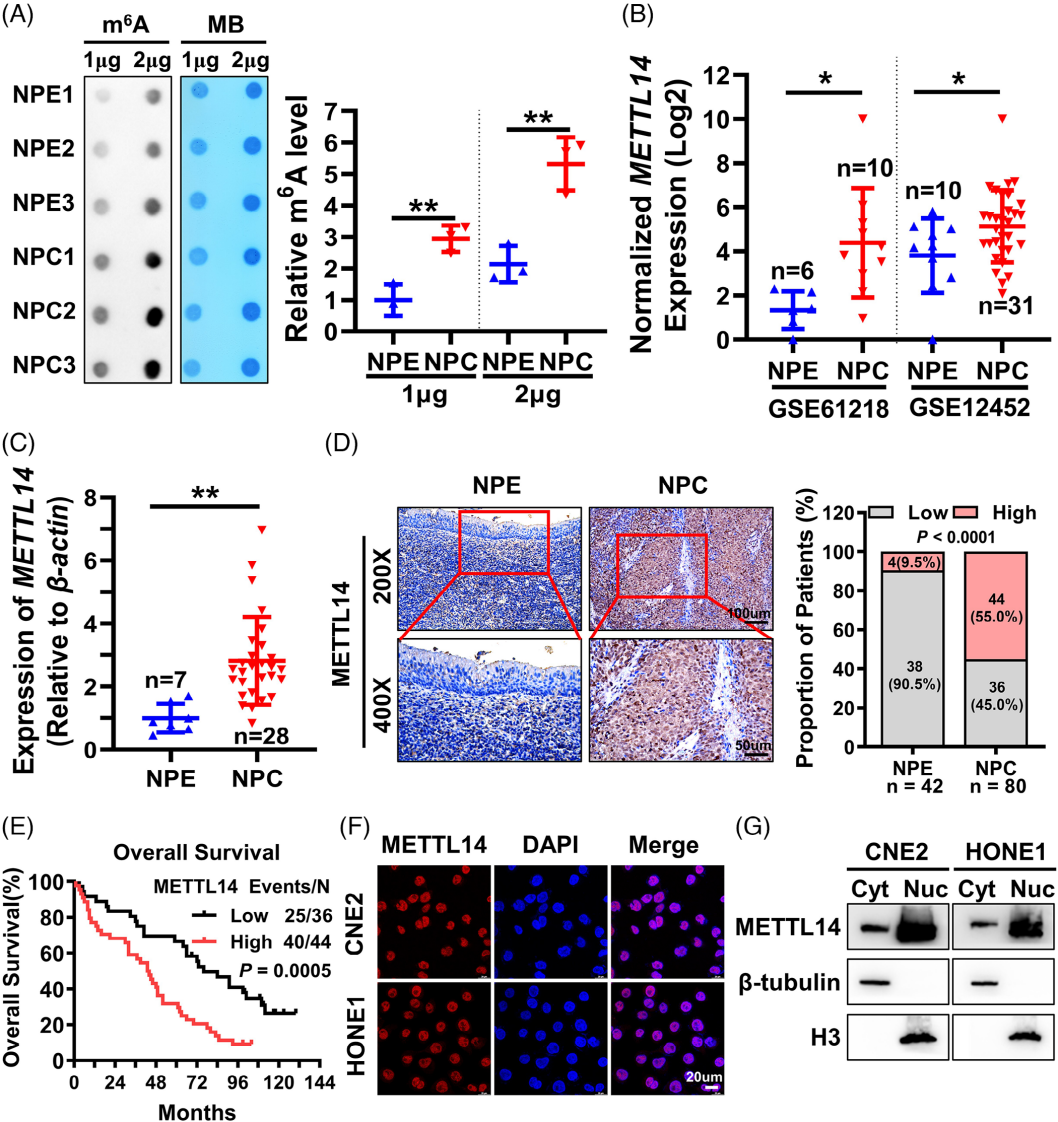
METTL14 is highly expressed in NPC and associated with poor prognosis of patients. (A) RNA m^6^A dot blot assays were employed to assess the m^6^A levels of total RNA in three non‐tumour nasopharyngeal epithelial (NPE) tissues and three NPC tissues (left panel), with greyscale scanning was conducted for statistical analysis (right panel). Methylene blue (MB) staining served as loading control. (B) METTL14 expression in NPC datasets GSE61218 and GSE12452. (C) METTL14 mRNA expression levels were detected in 28 NPC and 7 non‐tumour NPE tissues by RT‐qPCR. (D) IHC assays were applied to detect the expression of METTL14 protein in 80 NPC and 42 adjacent non‐tumour NPE tissues. Scale bars: 200 ×, 100 µm; 400 ×, 50 µm. Left panel: Representative images of METTL14 expression in NPC and NPE tissues. Right panel: Statistical analysis of METTL14 expression in NPC and NPE tissues. (E) Kaplan−Meier overall survival (OS) was conducted for METTL14 expression in 80 patients with NPC. (F) IF assays were applied to detect the METTL14 localization in CNE2 and HONE1 cells. Cell nuclei were counterstained with DAPI (blue). Scale bar: 20 µm. (G) Protein nuclear‐cytoplasmic fractionation assays were performed to assess the METTL14 localization in CNE2 and HONE1 cells. H3 was used as a nuclear marker, and β‐tubulin was used as a cytoplasmic marker. Cyt, cytoplasm; Nuc, nucleus. Data were presented as mean ± SD. *p < 0.05; ***p < 0.001.

To explore potential changes in m^6^A modification levels in NPC, we initially employed the m^6^A dot blot method to assess total RNA m^6^A modification levels in three NPC tissue samples and three epithelial tissues from chronic rhinitis patients (utilized as normal controls). Notably, we observed a substantial elevation in RNA m^6^A modification levels in NPC compared to non‐tumour NPE tissues (Figure 1A). This finding suggests that aberrant m^6^A modification might play a role in NPC development, and the increased m^6^A levels in NPC could be linked to the crucial involvement of m^6^A methyltransferases and m^6^A demethylases. We further analysed the expression of m^6^A methyltransferases (METTL14, WTAP, METTL3) and m^6^A demethylases (FTO, ALKBH5) in NPC using the GEO datasets GSE12452 and GSE61218. Our results revealed that WTAP mRNA was significantly upregulated in NPC tissues only in GSE61218, while METTL3 and FTO mRNA levels showed no significant difference, and ALKBH5 mRNA was only downregulated in NPC tissues in GSE12452 (Figure S1A). Notably, only METTL14 mRNA showed significant upregulation in NPC tissues in both datasets (Figure 1B). To further confirm the expression of METTL14, WTAP, METTL3, FTO and ALKBH5 in NPC tissues, we utilized RT‐qPCR to detect their expression in 28 NPC tissues and 7 nasopharyngeal chronic inflammation tissues. The results showed that WTAP mRNA was upregulated in NPC tissues, as previously reported,^25^ METTL3 and FTO showed no differential expression, while ALKBH5 was downregulated in NPC tissues (Figure S1B). Of note, METTL14 exhibited the most significant differential expression, being upregulated in NPC tissues (Figure 1C) with no relevant reports to date.

IHC performed on paraffin sections of 80 NPC and 42 adjacent non‐tumour NPE tissues demonstrated elevated METTL14 protein expression in NPC tissues (Figure 1D). Importantly, the heightened expression of METTL14 correlated positively with a poorer prognosis in NPC patients (Figure 1E). Immunofluorescence (IF) and nuclear‐cytoplasmic fractionation assays illustrated the predominant localization of METTL14 in the nucleus (Figure 1F,G). Collectively, these findings suggest that the increased expression of METTL14 may significantly contribute to the elevation in m^6^A levels in NPC, potentially fostering the development of NPC through m^6^A modification.

### 3.2 METTL14 promotes the proliferation and metastasis of NPC cells in vitro and in vivo

**FIGURE 2:**
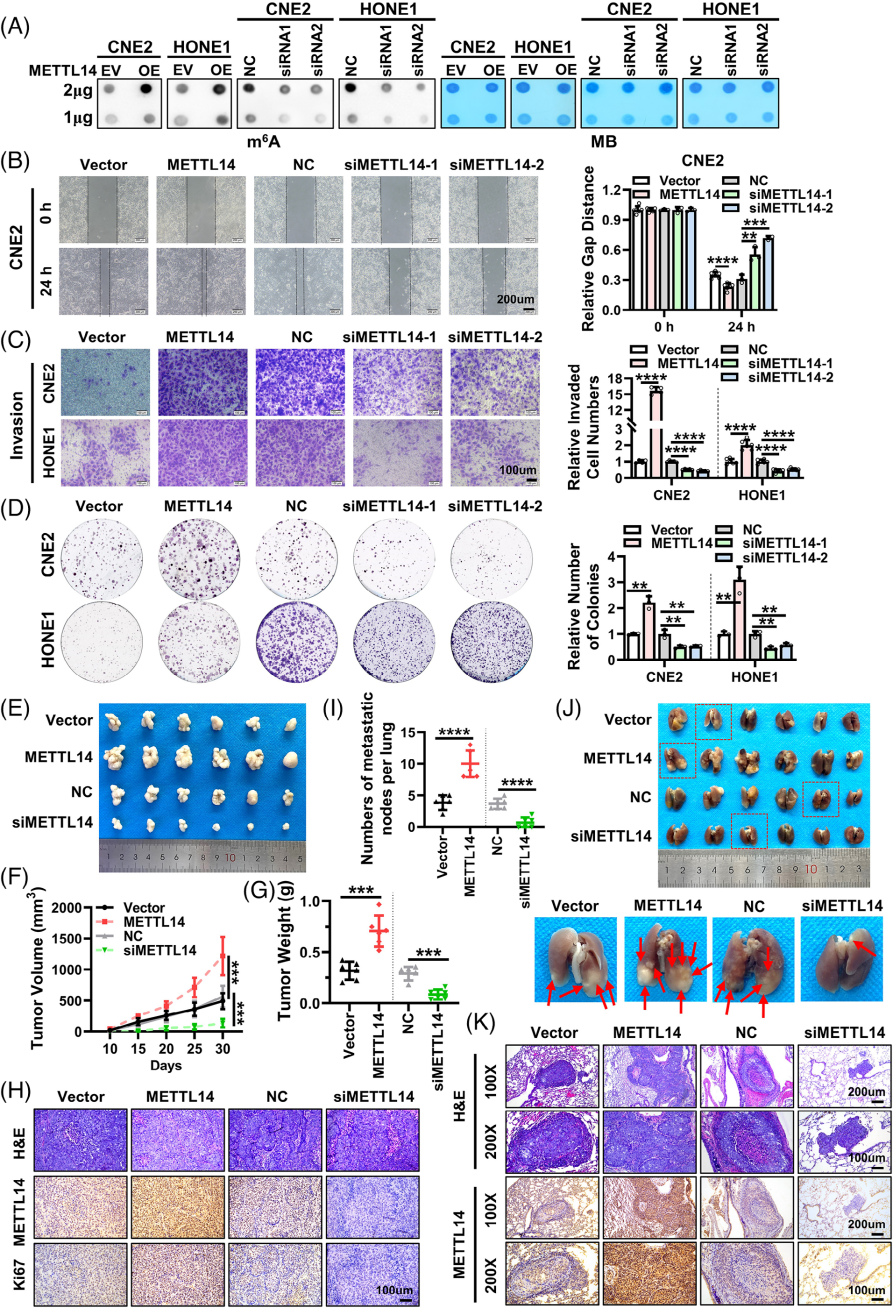
METTL14 promotes the proliferation and metastasis of NPC cells in vitro and in vivo. (A) RNA m^6^A dot blot assays were performed to detect the m^6^A levels of total RNA after overexpression or knockdown of METTL14 in CNE2 and HONE1 cells. Methylene blue (MB) staining served as loading control. (B) Wound healing assays were used to assess cell migration after overexpression or knockdown of METTL14 in CNE2 cells. Representative images (left panel) and statistical analysis (right panel) were presented. Images were acquired at 0 and 24 h. Scale bar: 200 µm. (C) Transwell invasion assays were performed to examine cell invasion ability after overexpression or knockdown of METTL14 in CNE2 and HONE1 cells. Representative images (left panel) and statistical analysis (right panel) were presented. Scale bar: 100 µm. (D) Colony formation assays were conducted to assess cell proliferation after overexpression or knockdown of METTL14 in CNE2 and HONE1 cells. (E) Images of subcutaneous tumours in nude mice (*n* = 6 per group). (F, G) Subcutaneous tumour volume growth curve (F) and tumour weight (G) in nude mice. (H) Representative images of subcutaneous tumour sections for H&E staining and IHC detection of METTL14 and Ki67 expression. Scale bar: 200 ×, 100 µm. (I) Quantification of the number of metastatic nodules on lung surface. (J) Representative images of lung tissue from nude mouse (upper panel, *n* = 6 per group). Arrows indicate metastatic nodules on the lung surface (lower panel). (K) Representative images of H&E‐stained lung metastatic tumour foci and METTL14 expression by assayed IHC. Scale bars: 100 ×, 200 µm; 200 ×, 100 µm. Data were presented as mean ± SD. **p < 0.01; ***p < 0.001; ****p < 0.0001.

In order to investigate the involvement of METTL14 in NPC development, we designed and synthesized two siRNA sequences (siMETTL14‐1, siMETTL14‐2) to downregulate METTL14 expression in NPC cells. Conversely, we utilized a METTL14 overexpression vector (pcDNA3.0‐METTL14) to induce METTL14 overexpression (Figure S2A,B). m^6^A dot blot assays confirmed that siRNA‐mediated knockdown of METTL14 significantly reduced RNA m^6^A levels in NPC cells, whereas METTL14 overexpression elevated cellular RNA m^6^A levels (Figure 2A). Wound healing assays showed that METTL14 knockdown markedly impeded the migration of NPC cells, while METTL14 overexpression significantly enhanced cell migration (Figure 2B and Figure S2C). Transwell assays further validated that METTL14 knockdown attenuated the migration and invasion capabilities of NPC cells, whereas METTL14 overexpression increased their migration and invasion abilities (Figure 2C and Figure S2D). Furthermore, we observed that METTL14 knockdown led to a reduction in the proliferation and colony formation capacity of NPC cells, whereas METTL14 overexpression exerted opposite effects (Figure S2E and Figure 2D). Altogether, these results strongly indicate that METTL14 plays a pivotal role in promoting NPC cells proliferation, migration and invasion.

We established a subcutaneous tumour model and tail vein lung metastasis model in BALB/C nude mice using NPC cells with either METTL14 overexpression or knockdown to further assess the influence of METTL14 on NPC growth and metastasis in vivo. In the subcutaneous tumour model, METTL14 knockdown resulted in a delayed growth of xenograft tumours (Figure 2E). Compared to the control group, the METTL14 knockdown group exhibited significantly reduced tumour volume (Figure 2F) and weight (Figure 2G), while overexpression of METTL14 promoted the growth of the xenograft tumours (Figure 2E‐G). IHC staining revealed a significantly higher expression level of Ki67 in the METTL14 overexpression group in comparison with the METTL14 knockout group (Figure 2H). For the tail vein lung metastasis model, the METTL14 overexpression group displayed a significant increase in the number of lung metastatic nodules compared to the control group, while knocking down METTL14 significantly reduced the number of lung nodules (Figure 2I‐K). These findings collectively suggest that both in vitro and in vivo METTL14 promote NPC proliferation and metastasis.

### 3.3 METTL14 upregulates the expression of ANKRD22 to promote NPC malignant progression

**FIGURE 3:**
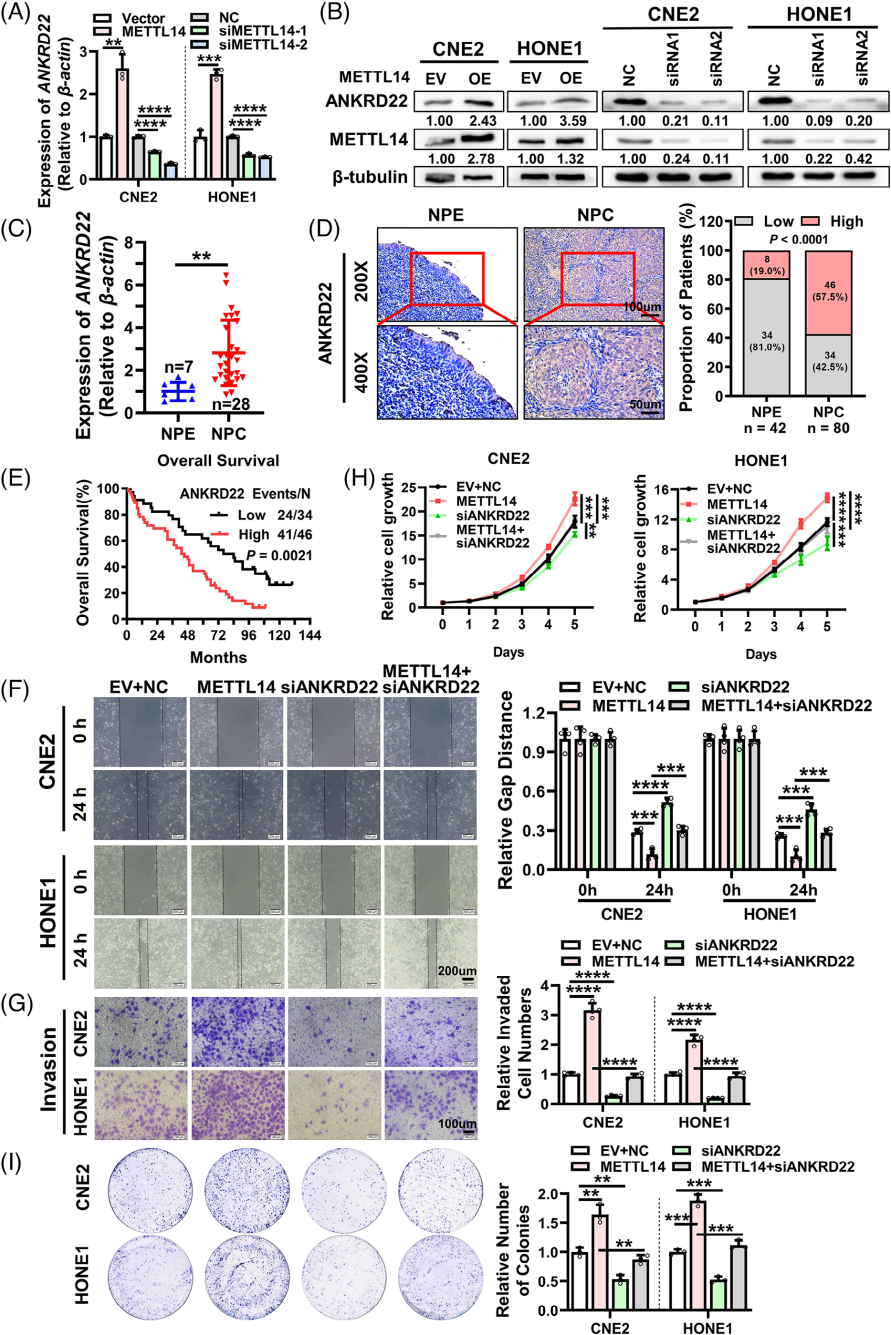
METTL14 upregulates the expression of ANKRD22 to promote NPC malignant progression. (A, B) The mRNA (A) and protein (B) levels of ANKRD22 after overexpression or knockdown of METTL14 in CNE2 and HONE1 cells as detected by RT‐qPCR and western blotting, respectively. (C) ANKRD22 mRNA expression levels in 28 NPC and 7 non‐tumour NPE tissues quantified by RT‐qPCR. (D) IHC assays were employed to determine the expression of ANKRD22 protein in 80 NPC and 42 adjacent non‐tumour NPE tissues. Scale bars: 200 ×, 50 µm; 400 ×, 20 µm. Left panel: Representative images of ANKRD22 expression in NPC and NPE tissues. Right panel: Statistical analysis of ANKRD22 expression in NPC and NPE tissues. (E) Kaplan−Meier overall survival (OS) was conducted for ANKRD22 expression in 80 patients with NPC. (F−I). Wound healing assays (F), Transwell invasion assays (G), MTT assays (H) and colony formation assays (I) were conducted to determine the effect of ANKRD22 on the METTL14‐mediated migration, invasion and proliferation of CNE2 and HONE1 cells. F, Scale bar: 200 µm. G, Scale bar: 100 µm. Data were presented as mean ± SD. **p < 0.01; ***p < 0.001; ****p < 0.0001.

To elucidate the potential mechanism underlying METTL14‐mediated promotion of NPC growth and metastasis, we conducted an analysis of m^6^A and gene expression profiling microarray data from clinical NPC tissue samples.^28^ This dataset encompassed measurements of both m^6^A modification levels and mRNA expression levels in NPC clinical samples. A total of 739 genes were identified as differentially expressed in NPC tissues, which also exhibited significant changes in m^6^A modification levels of their mRNA (|log_2_FC| ≥ 1, *p *< .05) (Figure S3A). Specifically, 92 genes demonstrated significant upregulation in both m^6^A modification status and mRNA expression in NPC tissues (Hyper‐up), while 647 genes displayed significant downregulation in both m^6^A modification status and mRNA expression (Hypo‐down) (Figure S3B and Table S7). Upregulation of m^6^A modification levels often leads to upregulation of gene expression levels in NPC,^27^, ^28^ and considering that METTL14 acts as an m^6^A‐modified writer that is upregulated in NPC, we performed pathway enrichment analysis of 92 genes (Hyper‐up) using Metascape,^29^ revealing enrichment primarily in signalling pathways such as cell cycle and fatty acid metabolism (Figure S3C). Since our goal was to identify downstream targets of METTL14, we selected the top 10 significantly upregulated at both m^6^A and mRNA levels (ANKRD22, EGFL6, ZIC2, LUZP2, SCNN1G, TMPRSS11F, TNFAIP6, CDC6, SUCNR1, WNT2) as candidate genes. Subsequently, we investigated the effect of METTL14 on their expressions. Knocking down METTL14 significantly decreased the mRNA expression of ANKRD22 in NPC cells, while overexpression of METTL14 significantly increased the mRNA expression of ANKRD22 (Figure 3A). In contrast, the expression changes of other genes were not as pronounced (Figure S3D). Consistent with the alterations observed at the RNA level, knocking down METTL14 reduced the protein expression of ANKRD22, while overexpression of METTL14 elevated the protein level of ANKRD22 (Figure 3B). Further analysis using RT‐qPCR on chronic inflammation tissues of the nasopharynx and NPC tissues revealed that ANKRD22 mRNA expression was higher in NPC tissues (Figure 3C). IHC results showed that, compared to adjacent non‐tumour NPE, ANKRD22 protein was highly expressed in NPC tissues (Figure 3D). Additionally, patients with high expression of ANKRD22 in NPC exhibited poor overall survival (Figure 3E). Moreover, ANKRD22 expression in NPC tissues was also markedly positively correlated with METTL14 (Figure S3E,F). These findings collectively suggest that METTL14 upregulates the expression of ANKRD22 in NPC.

To probe into the biological role of ANKRD22 in NPC, we employed siRNAs targeting ANKRD22 to knock down its expression and utilized an overexpression vector (pcDNA3.1‐ANKRD22) to elevate ANKRD22 expression. RT‐qPCR and western blotting confirmed the efficiency of knockdown and overexpression in NPC cell lines (Figure S3G,H). Overexpression of ANKRD22 promoted the migration (Figure S3I,J), invasion (Figure S3J), proliferation (Figure S3K) and colony formation capability (Figure S3L) of NPC cells, while knockdown of ANKRD22 significantly inhibited NPC cells migration, invasion, proliferation and colony formation capability (Figure S3 I, J, K, L), aligning with the phenotypes observed with METTL14 overexpression or knockdown. Furthermore, in NPC cells, concurrent overexpression of METTL14 and knockdown of ANKRD22 revealed that knockdown of ANKRD22 attenuated the METTL14‐induced migration (Figure 3F and Figure S4A) and invasion (Figure 3G) abilities of NPC cells. MTT and colony formation assays indicated that ANKRD22 knockdown inhibited the proliferation (Figure 3H) and colony formation capability (Figure 3I) of METTL14‐overexpressing NPC cells. Conversely, simultaneous knockdown of METTL14 and overexpression of ANKRD22 in NPC cells showed that overexpression of ANKRD22 rescued the decreased cell migration (Figure S4B,C), invasion (Figure S4C), colony formation (Figure S4D) and proliferation (Figure S4E) abilities induced by METTL14 knockdown. These results suggest that METTL14 accelerates the malignant progression of NPC via upregulating ANKRD22.

### 3.4 METTL14 enhances the stability and translation of ANKRD22 mRNA in an m^6^A‐dependent manner

**FIGURE 4:**
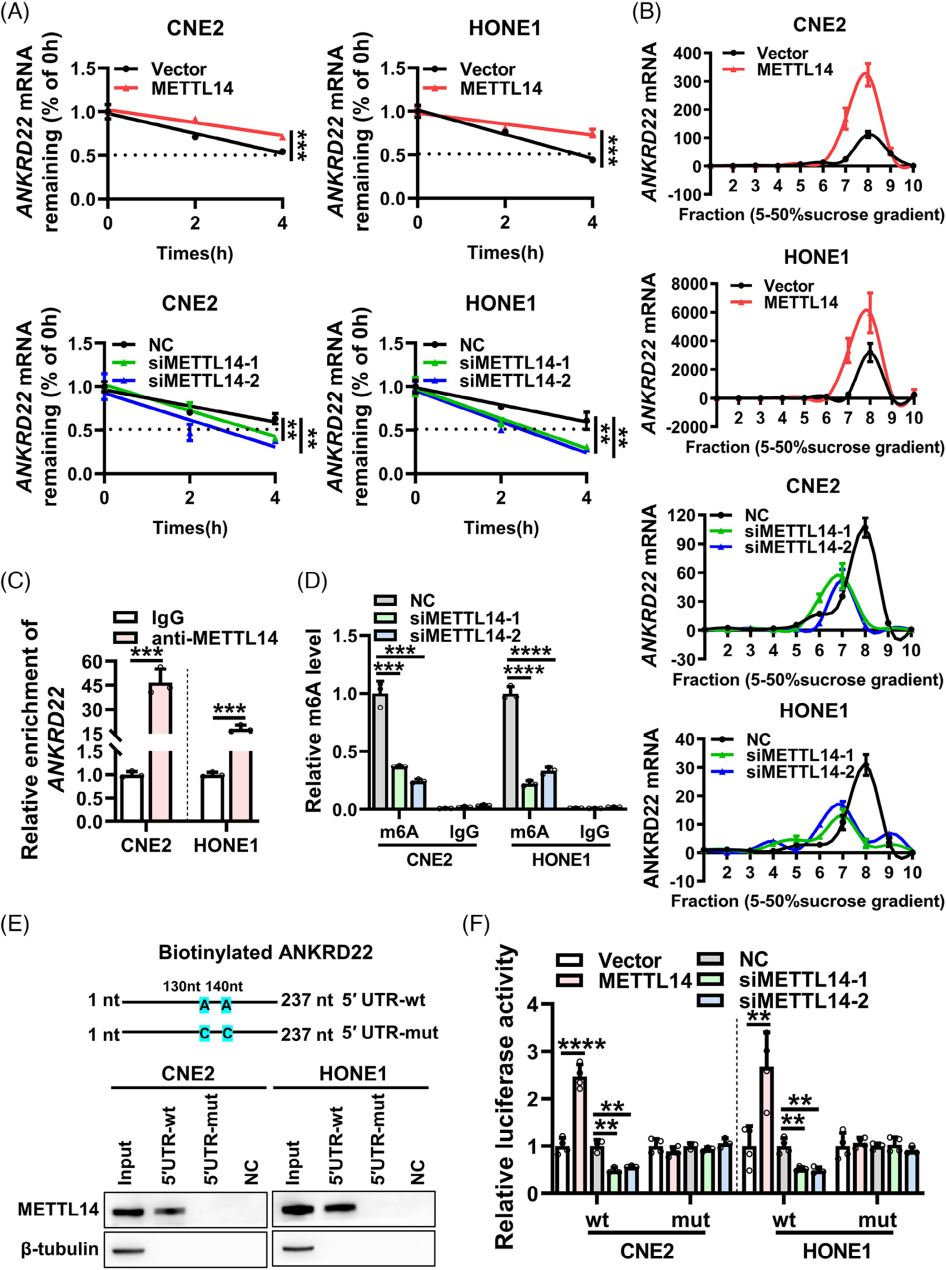
METTL14 enhances the stability and translation of ANKRD22 mRNA in an m^6^A‐dependent manner. (A) The ANKRD22 mRNA stability in CNE2 and HONE1 cells overexpression of METTL14 (upper panel) or knockdown of METTL14 (lower panel) was examined after treatment with actinomycin D (5 µg/mL) for the indicated times. (B) Polysome analysis was employed to determine the ANKRD22 mRNA translation efficiency after overexpression or knockdown of METTL14 in CNE2 and HONE1 cells. RNA from different ribosome fractions was extracted and analysed by RT‐qPCR. (C) RIP RT‐qPCR assays were performed to assess the binding of ANKRD22 mRNA with METTL14 in CNE2 and HONE1 cells. (D) MeRIP RT‐qPCR assays were conducted to assess the effect of knockdown of METTL14 on the m^6^A level of ANKRD22 mRNA (66−175 nt) in CNE2 and HONE1 cells. (E) RNA pull‐down assays were conducted to evaluate the interaction between METTL14 and ANKRD22 mRNA in CNE2 and HONE1 cells. NC, magnetic beads only. (F) Luciferase reporter assays were conducted to investigate the impact of METTL14 on the luciferase activity of the m^6^A sequence within the ANKRD22 5′ UTR in CNE2 and HONE1 cells. Data were presented as mean ± SD. **p < 0.01; ***p < 0.001; ****p < 0.0001.

To delve further into the mechanism by which METTL14 regulates ANKRD22 expression in NPC, we treated cells with the transcriptional inhibitor actinomycin D. It revealed that overexpression of METTL14 resulted in a slower degradation of ANKRD22 mRNA, while knockdown of METTL14 accelerated the degradation of ANKRD22 mRNA (Figure 4A). Additionally, polysome analysis demonstrated that overexpression of METTL14 increased the abundance of ANKRD22 mRNA in the translating pool, while knockdown of METTL14 led to a decrease in ANKRD22 mRNA in the translating pool, with no effect on the translation of the reference gene β‐actin (Figure 4B and Figure S5A). These findings suggest that METTL14 can also promote the translation of ANKRD22.

To test whether METTL14, a methyltransferase for m^6^A modification, regulates ANKRD22 mRNA through m^6^A modification, we searched potential m^6^A modification sites on ANKRD22 mRNA using two independent m^6^A databases (SRAMP,^30^ RMBase^31^) and combined the m^6^A modification sites shown by m^6^A and gene expression profiling microarray data.^28^ This analysis indicated that the 5′ UTR (at positions 130 and 140 nt) of ANKRD22 could be a potential target of METTL14 (Figure S5B).

To validate this finding, we conducted RNA immunoprecipitation (RIP) assays using an anti‐METTL14 antibody in CNE2 and HONE1 cells. The results revealed an enrichment of ANKRD22 mRNA by METTL14 (Figure 4C). Additionally, RNA pull‐down assays confirmed the direct interaction between ANKRD22 mRNA and METTL14 (Figure S5C). MeRIP RT‐qPCR further confirmed that the knockdown of METTL14 significantly reduced the m^6^A levels in the 5′ UTR of ANKRD22 mRNA in cells (Figure 4D). Additionally, RNA pull‐down assays demonstrated that mutation of the two m^6^A motifs in the ANKRD22 5′ UTR significantly diminished the specific binding of METTL14 to ANKRD22 transcripts (Figure 4E). Furthermore, mutation of the adenosine (A) to cytosine (C) in both m^6^A motifs in the 5′ UTR of ANKRD22, coupled with luciferase reporter assays, revealed that knockdown of METTL14 substantially reduced the luciferase activity from the wild‐type ANKRD22 5′ UTR. Conversely, overexpression of METTL14 significantly increased the luciferase activity from the wild‐type ANKRD22 5′ UTR. Notably, neither knockdown nor overexpression of METTL14 altered the luciferase activity from the mutant ANKRD22 5′ UTR (Figure 4F). These results collectively indicate that METTL14 interacts with the 5′ UTR region of ANKRD22, and through an m^6^A‐dependent pathway, METTL14 enhances the stability and translation of ANKRD22 mRNA in NPC cells.

### 3.5 IGF2BP2 recognizes METTL14‐mediated m^6^A modification on ANKRD22 mRNA

**FIGURE 5:**
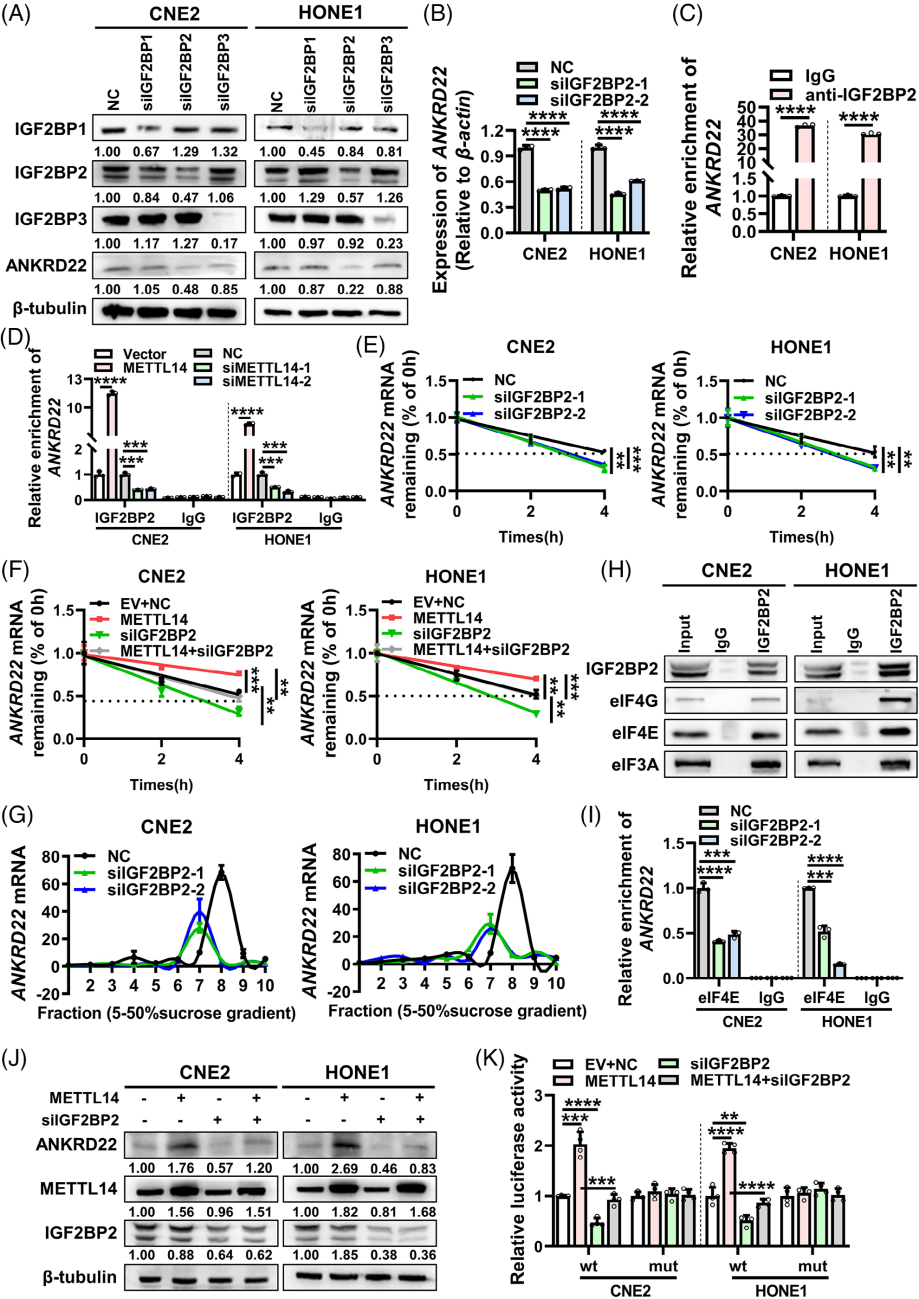
IGF2BP2 recognizes METTL14‐mediated m^6^A modification on ANKRD22 mRNA. (A) Protein levels of ANKRD22 after knockdown of IGF2BP1/2/3 in CNE2 and HONE1 cells were detected by western blotting. (B) RT‐qPCR assays were conducted to detect mRNA levels of ANKRD22 after knockdown of IGF2BP2 in CNE2 and HONE1 cells. (C) RIP RT‐qPCR assays were conducted to assess ANKRD22 mRNA binding with IGF2BP2 in CNE2 and HONE1 cells. (D) RIP RT‐qPCR assays were employed to assess IGF2BP2 binding to ANKRD22 mRNA after overexpression or knockdown of METTL14 in CNE2 and HONE1 cells. (E) After treatment of CNE2 and HONE1 cells with actinomycin D (5 µg/mL) for the indicated times, the effect of knockdown of IGF2BP2 on ANKRD22 mRNA stability was examined in the cells. (F) After treatment of CNE2 and HONE1 cells with actinomycin D (5 µg/mL) for the indicated times, the effect of co‐transfection of METTL14 overexpression vector and IGF2BP2 siRNA in cells on the stability of ANKRD22 mRNA was examined. (G) Polysome analysis was performed to assess the effect of knockdown of IGF2BP2 on the translation efficiency of ANKRD22 mRNA in CNE2 and HONE1 cells. RNAs in different ribosome fractions were extracted and analysed by RT‐qPCR. (H) IP assays were performed to analyse the binding of IGF2BP2 to eIF4G, eIF4E and eIF3A in CNE2 and HONE1 cells. (I) RIP RT‐qPCR assays were performed to assess the binding of eIF4E to ANKRD22 mRNA after knockdown of IGF2BP2 in CNE2 and HONE1 cells. (J) Protein levels of ANKRD22 after co‐transfection of METTL14 overexpression vector and IGF2BP2 siRNA in CNE2 and HONE1 cells were detected by western blotting. (K) Luciferase reporter assays were employed to evaluate the luciferase activity of the m^6^A sequence in the 5′ UTR of ANKRD22 after co‐transfection of METT14 overexpression vector and IGF2BP2 siRNA in CNE2 and HONE1 cells. Data were presented as mean ± SD. **p < .01; ***p < .001; ****p < .0001.

m^6^A modification on mRNA is recognized and bound by m^6^A reader proteins, which determine the fate of methylated mRNA. Two major families of readers that recognize m^6^A‐modified mRNA have been reported: YTHDF1/2/3 and IGF2BP1/2/3.^32^, ^33^, ^34^ In our study, we individually knocked down YTHDF1/2/3 and IGF2BP1/2/3 in two NPC cell lines and observed that only knockdown of IGF2BP2 significantly inhibited the protein level of ANKRD22 (Figure 5A and Figure S6A). Furthermore, the knockdown of IGF2BP2 also decreased the level of ANKRD22 mRNA (Figure 5B). RIP RT‐qPCR confirmed the binding between IGF2BP2 protein and ANKRD22 mRNA (Figure 5C). Consistently, RNA pull‐down assays showed that IGF2BP2 was significantly enriched by biotin‐labelled full‐length ANKRD22 mRNA (Figure S6B). Additionally, after inhibition of METTL14, the enrichment of ANKRD22 mRNA by anti‐IGF2BP2‐specific antibody was significantly impaired, while overexpression of METTL14 significantly increased this binding efficiency (Figure 5D). Nuclear‐cytoplasmic fractionation and IF assays showed that IGF2BP2 was mainly localized in the cytoplasm (Figure S6C,D). These findings suggest that IGF2BP2 may serve as an m^6^A reader for METTL14‐mediated ANKRD22 m^6^A modification.

We subsequently treated the cells with actinomycin D. The degradation of ANKRD22 mRNA was significantly accelerated in IGF2BP2 knockdown NPC cells compared with control cells (Figure 5E), which inhibited the effect of METTL14 overexpression on the degradation rate of ANKRD22 mRNA (Figure 5F). Polysome analysis in cells with IGF2BP2 knockdown showed a significant reduction of ANKRD22 mRNA in the translation pool, while β‐actin mRNA remained unchanged (Figure 5G and Figure S6E), indicating that IGF2BP2 promotes the translation of ANKRD22 mRNA. To understand the potential mechanism by which IGF2BP2 promotes ANKRD22 mRNA translation, we performed immunoprecipitation (IP) and found that IGF2BP2 physically interacted with eukaryotic initiation factors such as eIF4E, eIF4G and eIF3A (Figure 5H). On the other hand, RIP RT‐qPCR results revealed the IGF2BP2 knockdown dramatically suppressed the binding of eIF4E, eIF4G and eIF3A to ANKRD22 mRNA (Figure 5I and Figure S6F). These results suggest that IGF2BP2 may recruit eukaryotic initiation factors to ANKRD22 mRNA to enhance its translation. Furthermore, luciferase reporter assays revealed that knockdown of IGF2BP2 reduced the luciferase activity from the wild‐type ANKRD22 5′ UTR, while it had no effect on the luciferase activity from the m^6^A mutant of ANKRD22 5′ UTR (Figure S6G). These data indicate that IGF2BP2 enhances the stability and translation of ANKRD22 mRNA by recognizing the m^6^A site on ANKRD22 mRNA. Moreover, the knockdown of IGF2BP2 attenuated the effects of METTL14 on the levels of ANKRD22 mRNA (Figure S6H), protein (Figure 5J) and the luciferase activity from ANKRD22 5′ UTR (Figure 5K). Altogether, these findings suggest that IGF2BP2 enhances the stability and translational efficiency of ANKRD22 mRNA by recognizing and binding to the METTL14‐mediated m^6^A modification site on ANKRD22 mRNA.

### 3.6 METTL14 prompts lipid metabolism in NPC cells by upregulating ANKRD22 expression

**FIGURE 6:**
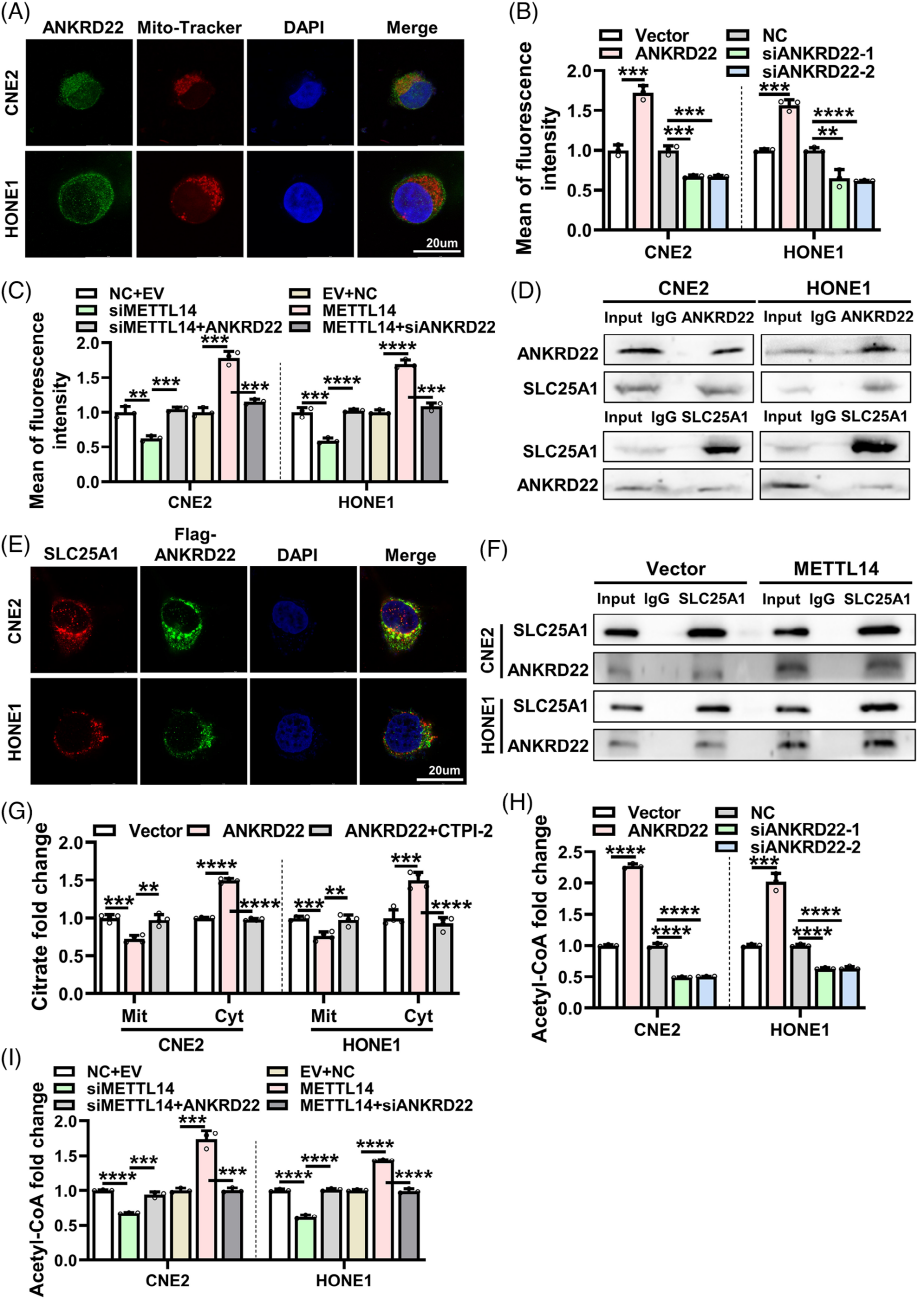
METTL14 prompts lipid metabolism in NPC cells by upregulating ANKRD22 expression. (A) IF assays showed co‐localization of ANKRD22 (green) and mitochondria (red) in the cytoplasm in CNE2 and HONE1 cells. Cell nuclei were counterstained with DAPI (blue). Scale bar: 20 µm. (B) Flow cytometry assaying lipid content after overexpression or knockdown of ANKRD22 in CNE2 and HONE1 cells. The original data are shown in Figure S7B. (C) Flow cytometry assays assaying lipid content after co‐transfection of METTL14 siRNA and ANKRD22 overexpression vector or co‐transfection of METTL14 overexpression vector and ANKRD22 siRNA in CNE2 and HONE1 cells. The original data are shown in Figures S7D and E. (D) Co‐IP assays analysing the interaction between ANKRD22 and SLC25A1 in CNE2 and HONE1 cells using anti‐ANKRD22 antibody (upper panel) or anti‐SLC25A1 antibody (lower panel). (E) IF assays showing co‐localization of SLC25A1 (red) and Flag‐ANKRD22 (green) in the cytoplasm in CNE2 and HONE1 cells. Cell nuclei were counterstained with DAPI (blue). Scale bar: 20 µm. (F) Interaction between ANKRD22 and SLC25A1 after overexpression of METTL14 in CNE2 and HONE1 cells was detected by IP using anti‐SLC25A1 antibody. (G) Citrate assay kit was used to detect the effect of overexpression of ANKRD22 and treatment with CTPI‐2 on citrate contents in mitochondria and cytoplasm in CNE2 and HONE1 cells. Cyt, cytoplasm; Mit, mitochondrion. (H) Acetyl‐CoA assay kit was employed to measure acetyl‐CoA content after overexpression or knockdown of ANKRD22 in CNE2 and HONE1 cells. (I) Acetyl‐CoA assay kit was employed to measure acetyl‐CoA contents in CNE2 and HONE1 cells after co‐transfecting with METTL14 siRNA and ANKRD22 overexpression vector or co‐transfecting with METTL14 overexpression vector and ANKRD22 siRNA. Data were presented as mean ± SD. **p < .01; ***p < .001; ****p < .0001.

ANKRD22, a protein featuring four ankyrin repeat (ANKR) motifs, has been implicated in the development of various cancers.^35^, ^36^, ^37^ Studies have suggested that ANKRD22 is localized to mitochondria and may be associated with lipid metabolism reprogramming.^38^ We conducted gene set enrichment analysis (GSEA) using the GEO database GSE12452 dataset and found that the expression of genes related to lipid metabolism and ANKRD22 expression in NPC correlated positively (Figure S7A−F). Western blotting analysis of cytoplasmic and mitochondrial fractions isolated from NPC cells further substantiated that ANKRD22 was predominantly localized to mitochondria (Figure S8A). IF assays demonstrated the co‐localization of ANKRD22 with mitochondria (Figure 6A). Flow cytometry and IF assays revealed that overexpression of ANKRD22 increased the lipid content in cells (Figure 6B and Figure S8B,C) and counteracted the effect of METTL14 knockdown on lipid content in NPC cells (Figure 6C and Figure S8D). Conversely, the knockdown of ANKRD22 reduced the lipid content in NPC cells (Figure 6B and Figure S8B,C) and mitigated the effect of METTL14 overexpression on lipid content (Figure 6C and Figure S8E). These results suggest that METTL14 may promote lipid metabolism in NPC cells by upregulating ANKRD22.

The ANKR motif within ANKRD22 is known for its specific mediation of protein−protein interactions.^39^ To further explore the mechanism through which ANKRD22 promotes lipid metabolism in NPC cells, we investigated whether ANKRD22 interacts with key enzymes on the mitochondria to facilitate lipid synthesis. We performed Co‐IP assays to analyse the binding of ANKRD22 and key enzymes of lipid metabolism (SLC25A1, ACC1, ACLY, FASN, SCD1), and the results showed that ANKRD22 significantly bound only to SLC25A1 (Figure S8F). The mitochondrial citrate/isocitrate carrier (CIC) SLC25A1 plays a pivotal role in exporting citrate from the mitochondria to the cytoplasm, thereby promoting de novo lipogenesis.^40^, ^41^ Endogenous Co‐IP assays also revealed the interaction between ANKRD22 and SLC25A1 (Figure 6D). IF assays further demonstrated the co‐localization of SLC25A1 and ANKRD22 proteins on mitochondrial (Figure 6E). To determine the specific interaction regions between ANKRD22 and SLC25A1, Co‐IP assays showed that the ANKR3 domain of ANKRD22 and the Solcar2 domain of SLC25A1 are essential for their binding (Figure S8G,H). Moreover, Co‐IP assays showed that METTL14 promotes the binding of ANKRD22 to SLC25A1 (Figure 6F). Overexpression of ANKRD22 increased the cytoplasmic citrate content (Figure 6G), and the addition of the SLC25A1‐specific inhibitor compound CTPI‐2 reversed the effect of ANKRD22 on citrate content (Figure 6G). Citrate serves as a source of acetyl‐CoA for fatty acid synthesis in the cytoplasm. Of note, overexpression of ANKRD22 increased the levels of acetyl‐CoA in CNE2 and HONE1 cells (Figure 6H) and mitigated the effect of siMETTL14 on cytoplasmic acetyl‐CoA levels (Figure 6I). Conversely, the knockdown of ANKRD22 decreased the abundance of acetyl‐CoA in cells (Figure 6H) and attenuated the effect of METTL14 overexpression on cellular acetyl‐CoA content (Figure 6I). These findings indicate that ANKRD22, through its interaction with SLC25A1, mediates the impact of METTL14 on lipid metabolism in NPC cells.

### 3.7 ANKRD22 activates downstream gene transcription through enhancing H3K27AC

**FIGURE 7:**
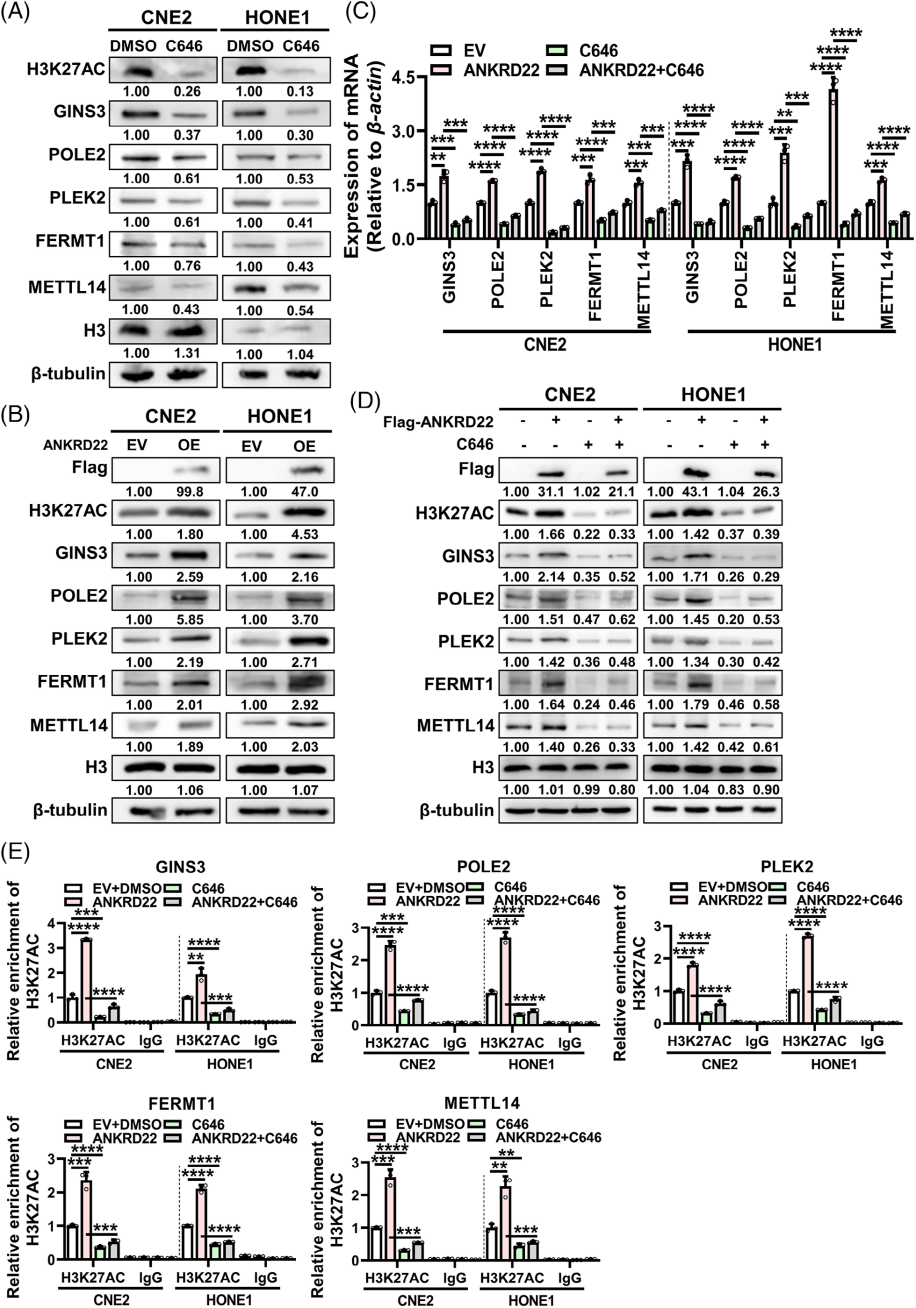
ANKRD22 activates downstream gene transcription through enhancing H3K27AC. (A) Protein levels of H3K27AC, GINS3, POLE2, PLEK2, FERMT1 and METTL14 in CNE2 and HONE1 cells after treatment with C646 (20 µM) were detected by western blotting. (B) Protein levels of H3K27AC, GINS3, POLE2, PLEK2, FERMT1 and METTL14 in CNE2 and HONE1 cells after overexpression of ANKRD22 were detected by western blotting. (C, D) GINS3, POLE2, PLEK2, FERMT1 and METTL14 mRNA (C) and protein (D) levels in CNE2 and HONE1 cells after overexpression of ANKRD22 and treatment with C646 (20 µM) were detected by RT‐qPCR and western blotting, respectively. (E) ChIP‐qPCR assays evaluating H3K27AC enrichment near the promoters of GINS3, POLE2, PLEK2, FERMT1 and METTL14 in CNE2 and HONE1 cells after overexpression of ANKRD22 and treatment with C646 (20 µM). Data were presented as mean ± SD. **p < .01; ***p < .001; ****p < .0001.

Acetyl‐CoA serves as a central metabolic intermediate, acting as a key precursor for lipid production and the sole donor of acetyl groups for intracellular histone acetylation.^42^, ^43^ Histone acetylation, exemplified by histone H3 lysine 27 acetylation (H3K27AC), is a well‐established marker of active chromatin associated with gene expression.^44^, ^45^ Analysis of the gene microarray dataset GSE12452 identified two molecules related to the cell skeleton, PLEK2 and FERMT1, as well as two cell cycle‐related molecules, GINS3 and POLE2, displaying strong correlations with ANKRD22 (Figure S9A,B and Table S8). These four proteins exhibited elevated expression in NPC tissues (Figure S9C). Chromatin immunoprecipitation (ChIP)‐seq data for H3K27AC in NPC cell lines (C666‐1, HK1 and HNE1) from the ENCODE database^46^ (visualized through the UCSC browser) demonstrated high H3K27AC signals at the GINS3, POLE2, PLEK2 and FERMT1 genes (Figure S10), suggesting the importance of H3K27AC in driving the expression of these genes and promoting invasion, migration and proliferation in NPC. Additionally, the promoter region of METTL14 was also enriched with H3K27AC signals (Figure S10), potentially contributing to its high expression in NPC. Treatment of cells with the histone acetyltransferase inhibitor C646 significantly reduced H3K27AC (Figure 7A) and led to a significant decrease in the RNA levels of GINS3, POLE2, PLEK2, FERMT1 and METTL14 (Figure S9D) as well as their protein levels (Figure 7A). Overexpression of ANKRD22 enhanced H3K27AC in cells (Figure 7B) and upregulated the RNA and protein levels of GINS3, POLE2, PLEK2, FERMT1 and METTL14 (Figure S9E and Figure 7B). Notably, these effects were reversed when C646 was added (Figure 7C,D). Furthermore, ChIP‐qPCR assays confirmed that overexpression of ANKRD22 enhanced H3K27AC near the promoters of these genes (Figure S9F), while adding C646 attenuated the effect of ANKRD22 on H3K27AC of these genes (Figure 7E). These results demonstrate that the heightened H3K27AC signal induced by ANKRD22 overexpression can, on one hand, promote the increased expression of cytoskeleton‐related proteins (PLEK2, FERMT1) and cell cycle‐related proteins (GINS3, POLE2), thereby enhancing the invasion, migration and proliferation of NPC cells. On the other hand, it can also promote the transcription of METTL14, forming a feedback loop.

### 3.8 Positive correlations between METTL14 and ANKRD22 along with its downstream genes in NPC clinical samples

**FIGURE 8:**
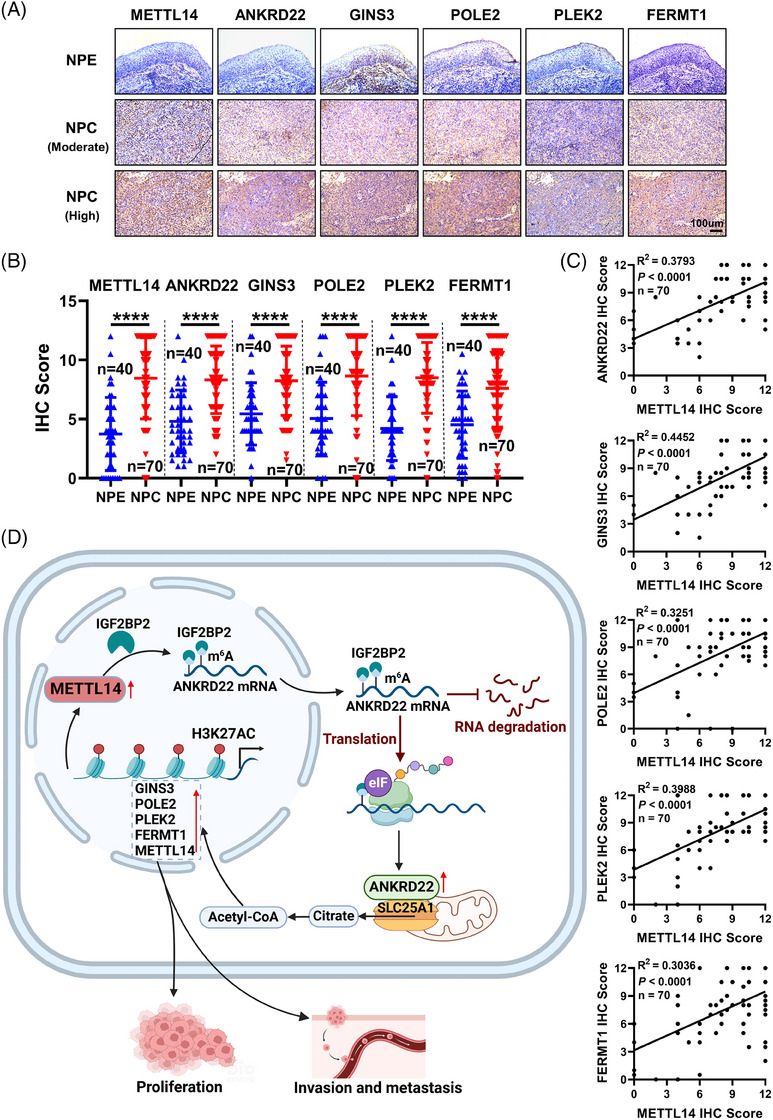
Positive correlations between METTL14 and ANKRD22 along with its downstream genes in NPC clinical samples. (A) IHC assays evaluating the expression levels of METTL14, ANKRD22, GINS3, POLE2, PLEK2 and FERMT1 proteins in 70 NPC tissues and 40 adjacent NPE tissues. Scale bar: 200 ×, 100 µm. (B) Statistical analysis of METTL14, ANKRD22, GINS3, POLE2, PLEK2 and FERMT1 expression in 70 NPC tissues and 40 adjacent NPE tissues. (C) Correlation between METTL14 and ANKRD22, GINS3, POLE2, PLEK2, FERMT1 in 70 NPC tissues. (D) Diagram illustrating the mechanism by which METTL14 promotes malignant progression of NPC through upregulation of m^6^A modification of ANKRD22. Data were presented as mean ± SD. ****p < .0001.

To further elucidate the role of METTL14 in promoting the malignant progression of NPC, we examined the expression of PLIN3 (a lipid droplet marker) in subcutaneous tumours (Figure S11A) and lung metastatic tissues (Figure S11B), and IHC data confirmed that lipid levels were higher in the METTL14 overexpression group and lower in the METTL14 knockdown group compared to the control group. In addition, we assessed the expression of molecules such as ANKRD22, GINS3, POLE2, PLEK2, FERMT1 and METTL14 in subcutaneous tumours (Figure S11A) and lung metastatic tissues (Figure S11B). IHC data showed heightened expression levels of ANKRD22, GINS3, POLE2, PLEK2 and FERMT1 in the METTL14 overexpression group and diminished expression levels in the METTL14 knockdown group. Further validation was performed through IHC analysis of paraffin sections of 70 NPC and 40 adjacent non‐tumour NPE tissues (Figure 8A,B). In comparison to NPE tissues, the levels of METTL14, ANKRD22, GINS3, POLE2, PLEK2 and FERMT1 were upregulated in NPC tissues. Furthermore, there was a strong positive correlation between the expression of METTL14 and that of ANKRD22, GINS3, POLE2, PLEK2 and FERMT1 (Figure 8C). These findings emphasize the critical effect of METTL14 in facilitating the malignant progression of NPC.

## 4 DISCUSSION

This study unveils the pivotal role of METTL14‐mediated m^6^A modification in the progression of NPC. METTL14 facilitates the expression of ANKRD22 through the METTL14‐IGF2BP2‐ANKRD22 signalling axis. ANKRD22, in turn, interacts with the citrate transporter protein SLC25A1, elevating intracellular acetyl‐CoA levels. This increase promotes lipid synthesis in NPC cells, sustaining rapid proliferation and metastasis of cancer cells. Simultaneously, heightened acetyl‐CoA levels regulate epigenetic modifications, inducing high H3K27AC signals near the promoters of cell cycle and cytoskeleton‐related genes, thereby accelerating the proliferation and metastasis of NPC. Notably, we observed that elevated acetyl‐CoA levels also enhance H3K27AC near the promoter of METTL14, leading to an upregulation of METTL14 expression, thus forming a positive feedback loop (Figure 8D). This feedback loop further perpetuates the proliferation and metastasis of NPC.

Alterations in epigenetic modifications, encompassing DNA, histone and RNA modifications, are pivotal contributors to the dysregulation of gene expression and play crucial roles in tumorigenesis. In recent years, the significance of RNA modifications in cancer has garnered attention, with the discovery of over 100 types of RNA modifications.^47^ Among these modifications, m^6^A modification stands out as the most abundant and highly conserved modification on transcripts, characterized by the consensus sequence [G/A/U] [G  >  A] m^6^A C [U  >  A  >  C].^48^, ^49^ It is prevalent in non‐coding regions such as the 5′ UTR^50^, ^51^ and 3′ UTR,^52^, ^53^ as well as in the coding sequence (CDS).^54^, ^55^ The m^6^A modification is dynamically regulated by methyltransferase writers, demethylase erasers, and subsequently recognized by readers. This intricate process determines the fate of numerous RNA molecules, thereby influencing dysregulated gene expression and impacting tumour progression.^56^, ^57^, ^58^

METTL14, a key component of the m^6^A methyltransferase complex, has been implicated in various cancers, exerting a significant role in tumour progression. For instance, in pancreatic cancer, METTL14 is upregulated, leading to decreased PERP levels through the m^6^A‐YTHDF2 axis, thereby promoting cancer growth and metastasis.^59^ In osteosarcoma, METTL14, highly expressed, prevents the degradation and enhances the translational efficiency of MN1 mRNA through the m^6^A‐IGF2BP2 axis, contributing to tumorigenicity and conferring all‐trans‐retinoic acid resistance.^60^ While prior studies have reported that m^6^A modification levels mediated by the methyltransferases WTAP and METTL3 can promote NPC development,^25^, ^26^, ^27^ the specific biological role and regulatory mechanism of METTL14 in NPC have not been elucidated to date. In this study, we observed a significant upregulation of METTL14 expression in NPC tissues, closely associated with poor prognosis in patients. Additionally, m^6^A dot blot assays revealed an overall increase in RNA m^6^A levels in NPC tissues compared to nasopharyngeal chronic inflammation tissues. We demonstrated that METTL14 promotes NPC cells proliferation and metastasis through in vitro and in vivo assays, indicating a close association between METTL14 expression and the progression of NPC. Leveraging the analysis of m^6^A and gene expression profiling microarray data,^28^ we identified candidate genes and confirmed in NPC cells that METTL14 positively regulated the RNA and protein levels of ANKRD22. Moreover, our findings indicate that METTL14 upregulates ANKRD22 expression, thereby promoting NPC malignant progression, establishing ANKRD22 as a crucial target gene of METTL14.

ANKRD22, situated on chromosome 10q23.31, is a protein featuring four ANKR motifs and has been implicated in the development of various tumours.^35^, ^36^, ^38^, ^61^, ^62^ Despite its reported involvement in tumorigenesis, the role of ANKRD22 in NPC remains unexplored. Our investigation unveiled that ANKRD22 plays a crucial role in promoting NPC cells proliferation, migration and invasion. Furthermore, as ANKRD22 is localized in the mitochondria,^38^ it implies a potential association with tumour metabolism. However, the regulatory mechanisms and functions of ANKRD22 in tumour cell metabolism reprogramming during tumorigenesis remain poorly understood. Our data analysis revealed a robust enrichment of gene sets associated with lipid metabolism reprogramming in NPC samples exhibiting high ANKRD22 expression. Experimental evidence further affirmed that the overexpression of ANKRD22 facilitates lipid metabolism reprogramming, augmenting cellular lipid content. These findings suggest that ANKRD22 influences NPC cells proliferation and metastasis through metabolic reprogramming. Notably, ANKRD22 also counteracted the reduction in lipid content in NPC cells mediated by METTL14 knockdown, indicating that METTL14 upregulates ANKRD22 expression to promote lipid metabolism in NPC cells.

Lipid metabolism reprogramming has emerged as a hallmark feature of cancer cells, playing a pivotal role in cancer progression. Lipids serve as essential components for cell membrane formation during rapid proliferation and act as an energy source, contributing to growth and metastasis. Cancer cells primarily obtain lipids through de novo synthesis and exogenous uptake. Previous studies have demonstrated the upregulation of enzymes involved in both fatty acid synthesis and catabolism, along with lipid accumulation, in various cancer types.^63^, ^64^ In lung adenocarcinoma, downregulation of fatty acid synthase ACLY reduced both proliferation and metastasis.^65^ In hepatocellular carcinoma, hepatic mTORC2 promoted de novo lipid synthesis, resulting in lipid deposition and tumour development.^66^ The mitochondrial CIC SLC25A1 plays a critical role by exporting citrate from the mitochondria to the cytoplasm. This action upregulates acetyl‐CoA synthesis, facilitating de novo lipid synthesis.^40^, ^41^ Acetyl‐CoA serves as a substrate not only for lipid synthesis but also for histone acetylation, a modification that promotes open chromatin conformation and enhances the expression of oncogenes, thereby fostering cancer growth and metastasis.^67^ Our study uncovered that the mitochondrial protein ANKRD22 interacts with SLC25A1, enhancing its citrate transport activity. This interaction leads to increased acetyl‐CoA levels and elevated H3K27AC. Importantly, the heightened H3K27AC levels upregulate the expression of cell cycle‐related genes (GINS3 and POLE2) and cytoskeleton‐related genes (PLEK2 and FERMT1), thereby promoting NPC cells proliferation, migration and invasion. Notably, the positive correlation between the expression of METTL14 and ANKRD22, GINS3, POLE2, PLEK2 and FERMT1 in NPC tissues underscores the clinical significance of the METTL14‐ANKRD22 axis in driving the growth and metastasis of NPC.

The analysis of clinical tissue samples from NPC revealed a substantial increase in both the RNA and protein levels of ANKRD22. To understand the impact of m^6^A modification on ANKRD22 expression, we explored two prominent m^6^A reader families, namely YTHDF1/2/3 and IGF2BP1/2/3.^32^, ^33^, ^34^ Our investigation uncovered that the depletion of IGF2BP2 significantly diminished both the RNA and protein levels of ANKRD22. Moreover, the absence of IGF2BP2 resulted in decreased stability and translation efficiency of ANKRD22 mRNA. This suggests that IGF2BP2 serves as a reader for METTL14‐mediated m^6^A modification on ANKRD22, thereby enhancing the stability and translation efficiency of ANKRD22 mRNA and contributing to its upregulation in NPC tissues.

Significantly, we observed heightened H3K27AC signals near the METTL14 promoter. Our data confirmed that the augmented H3K27AC, resulting from increased acetyl‐CoA content mediated by ANKRD22, facilitated METTL14 expression. This suggests the establishment of a positive feedback loop, METTL14‐m^6^A‐ANKRD22, potentially contributing to the elevated expression of METTL14 and high m^6^A levels in NPC. In our analysis of m^6^A and gene expression profiling microarray data,^28^ we verified ANKRD22 as a crucial target of METTL14. However, other genes also exhibited substantial upregulation in both m^6^A modification status and mRNA expression in NPC tissues (Hyper‐up). Our pathway enrichment analysis of the Hyper‐up genes showed that there are other signalling pathways besides lipid metabolism, such as mitotic G1 phase and G1/S transition, as well as apoptosis. These findings suggest that METTL14‐mediated m^6^A modification may regulate various signalling pathways that impact NPC development, warranting further investigation. Additionally, our findings indicate that METTL14 promotes lipid metabolism in NPC cells through ANKRD22 upregulation. Our results showed that METTL14 and ANKRD22 expression levels were upregulated in NPC tissues, and high expression of METTL14 and ANKRD22 was linked to a worse prognosis for the patients as indicated by survival analysis. This suggests that METTL14 and ANKRD22 could serve as potential new molecular markers for clinical adjuvant diagnosis and prognostic assessment of NPC. Furthermore, targeting METTL14, ANKRD22 and lipid metabolic pathways may represent novel therapeutic strategies for treating NPC. Studies have demonstrated the potent anti‐tumour activity of targeting lipid metabolism regulators.^68^ For instance, combining therapies targeting ANKRD22 with drugs that modulate lipid metabolism pathways, such as orlistat, holds promise in improving cancer treatment outcomes. However, further research and validation are necessary to explore these therapeutic approaches fully. Developing METTL14‐based inhibitors targeting ANKRD22 to impede lipid metabolism in tumour cells emerges as a promising strategy for future NPC treatment.

In summary, our findings elucidate a molecular mechanism wherein METTL14 facilitates lipid metabolism in NPC cells by elevating ANKRD22, establishing an aberrant regulatory loop that sustains m^6^A modifications in NPC cells. ANKRD22, through its impact on cellular lipid metabolism, raises intracellular acetyl‐CoA levels, enhances downstream H3K27AC gene expression, and ultimately contributes to tumorigenesis.

## 5 CONCLUSIONS

Our findings unveiled the vital function of METTL14 through the METTL14‐IGF2BP2‐ANKRD22 axis in the NPC progression. This study expands our comprehension of METTL14's role in NPC development, offering insights that can guide future research and exploration of therapeutic strategies for NPC.

## AUTHOR CONTRIBUTIONS

Lvyuan Li designed and completed most experiments. Qiling Tang, Junshang Ge, Dan Wang and Yijie Zhang performed some of the experiments. Yongzhen Mo, Yumin Wang and Qijia Yan collected tissue samples. Lvyuan Li analysed the data and wrote the manuscript. Fang Xiong, Qianjin Liao, Can Guo, Fuyan Wang, Ming Zhou, Bo Xiang, Zhaoyang Zeng, Lei Shi, Pan Chen and Wei Xiong revised the manuscript. Lei Shi, Pan Chen and Wei Xiong are responsible for research supervision and funding acquisition. All authors read and approved the final manuscript.

## CONFLICT OF INTEREST STATEMENT

The authors declare to have no conflict of interest.

## ETHICAL APPROVAL

The present study was approved by the Ethics Committee of Central South University.

## Supporting information

Supporting Information

Supporting Information

Supporting Information

Supporting Information

Supporting Information

Supporting Information

Supporting Information

Supporting Information

Supporting Information

## Data Availability

The data that support the findings of this study are available from the corresponding author upon reasonable request.
