# Supplementary material for: METTL14 promotes lipid metabolism reprogramming and sustains nasopharyngeal carcinoma progression via enhancing m6A modification of ANKRD22 mRNA
Source: Clin Transl Med. 2024 Jul 17;14(7):e1766. doi: 10.1002/ctm2.1766 (PMC11255023; doi:10.1002/ctm2.1766)
Supplement: Supplementary file 1 — Supporting Information [file CTM2-14-e1766-s003.docx]

Supplementary material

**
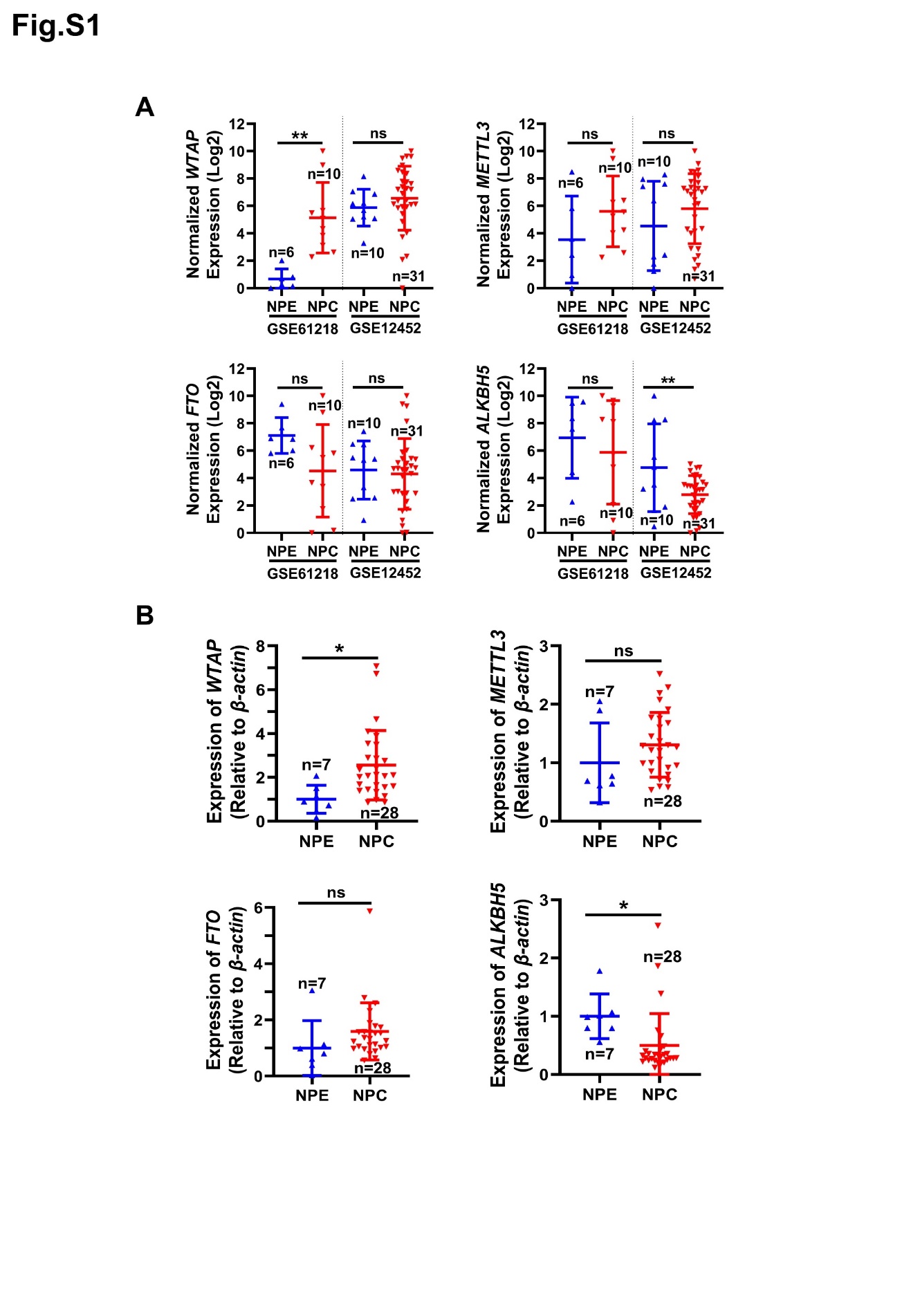
**

**Supplementary Fig. 1 NPC datasets and RT-qPCR analysis of the expression levels of WTAP, METTL3, FTO, and ALKBH5**

**A.** Expression of WTAP, METTL3, FTO, and ALKBH5 in NPC datasets GSE61218 and GSE12452.

**B.** WTAP, METTL3, FTO, and ALKBH5 mRNA expression levels were assessed in 28 NPC and 7 non-tumor NPE tissues by RT-qPCR.

Data were presented as the means ± SD. **p* < 0.05, ***p* < 0.01.

**
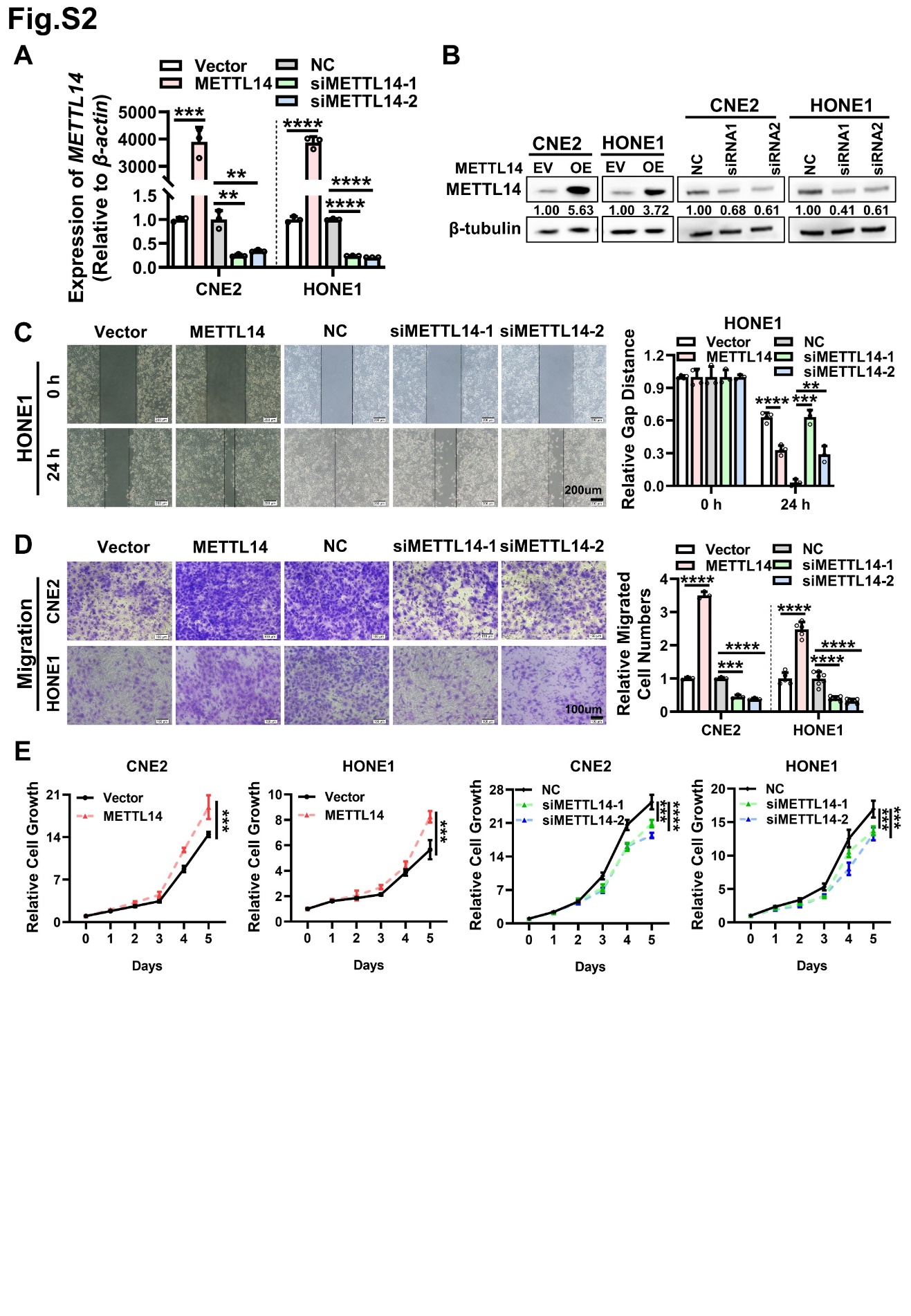
**

**Supplementary Fig. 2 METTL14 promotes the proliferation and migration of NPC cells in vitro**

**A, B.** Overexpression and knockdown of METTL14 were evaluated by RT-qPCR (**A**) and western blotting (**B**).

**C.** Wound healing assays were employed to evaluate cell migration ability after overexpression or knockdown of METTL14 in HONE1 cells. Representative images (left panel) and statistical analysis (right panel) were presented. Images were acquired at 0 and 24 hours. Scale bar: 200 μm.

**D.** Transwell migration assays were employed to evaluate cell migration ability after overexpression or knockdown of METTL14 in CNE2 and HONE1 cells. Representative images (left panel) and statistical analysis (right panel) were presented. Scale bar: 100 μm.

**E.** MTT assays were employed to evaluate cell proliferation ability after overexpression or knockdown of METTL14 in CNE2 and HONE1 cells.

Data were presented as mean ± SD. ***p* < 0.01, ****p* < 0.001, *****p* < 0.0001.


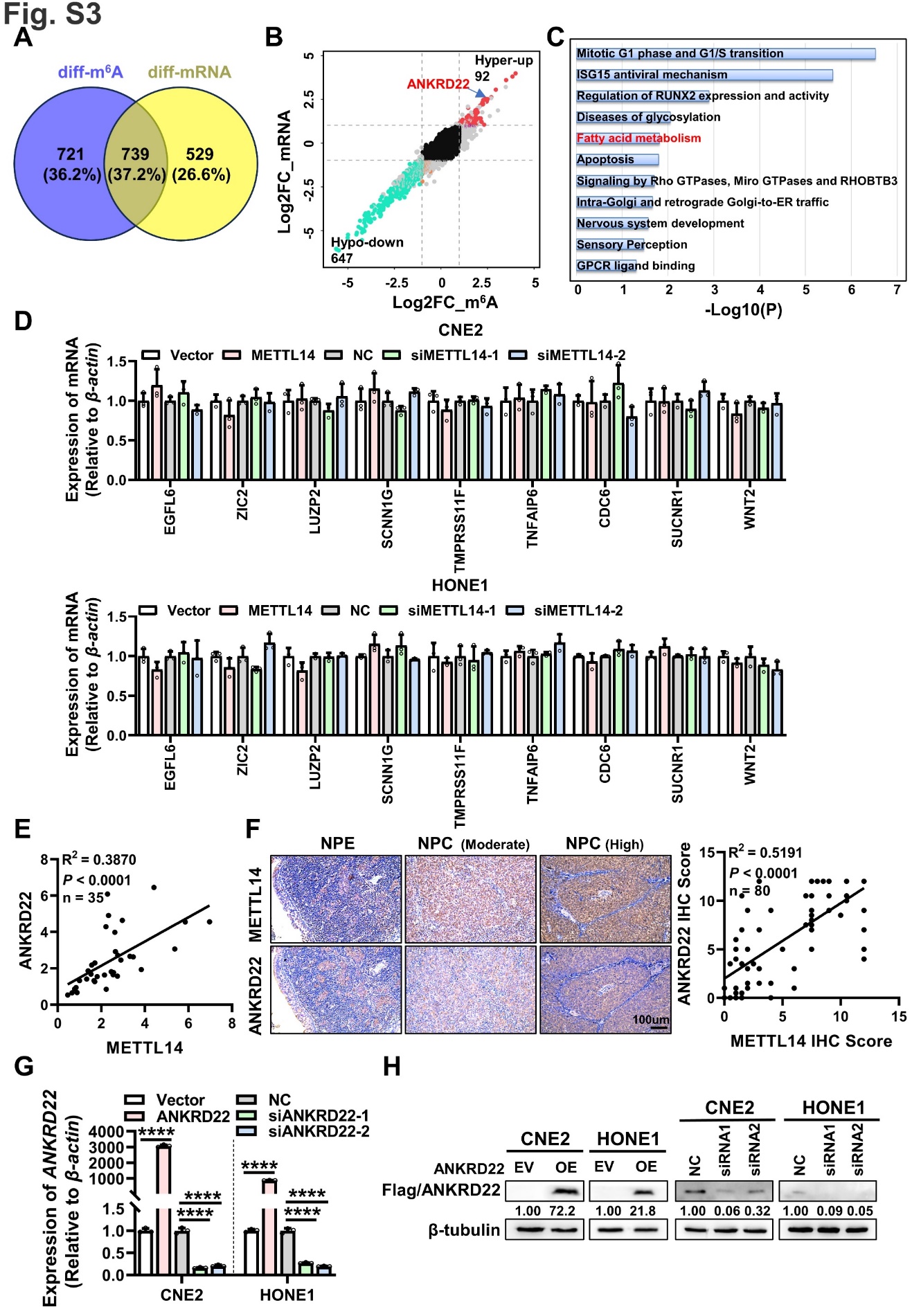


**
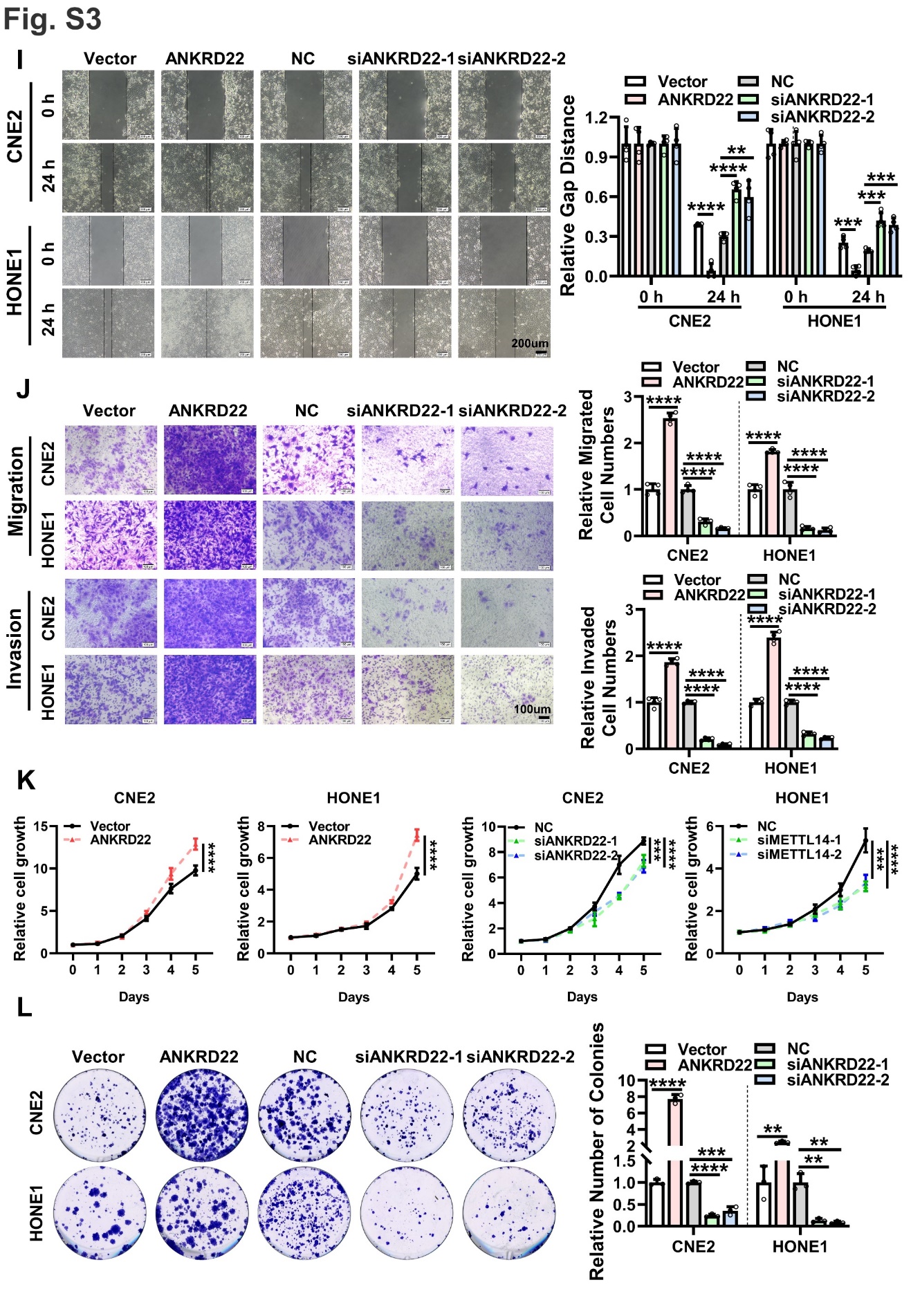
**

**Supplementary Fig. 3 ANKRD22 promotes proliferation, migration, and invasion of NPC cells in vitro.**

**A.** Venn diagram showing a total of 739 genes with abnormal m^6^A modification levels (1460 genes, |log_2_FC| ≥ 1, *p* < 0.05) as well as dysregulated mRNA expression (1268 genes, |log_2_FC| ≥ 1, *p* < 0.05), using NPE tissues as the control group.

**B.** The quadrantal diagram graph showing the distribution of 739 genes with significantly altered m^6^A modification levels and mRNA expression levels. Among them, 92 genes were significantly upregulated (Hyper-up) for both m^6^A modification and mRNA expression in NPC tissues, and 647 genes were significantly downregulated (Hypo-down) for both m^6^A modification and mRNA expression in NPC tissues.

**C.** Reactome pathway analysis of genes with upregulated m^6^A modification levels and differential expression in NPC.

**D.** EGFL6, ZIC2, LUZP2, SCNN1G, TMPRSS11F, TNFAIP6, CDC6, SUCNR1 and WNT2 mRNA levels were evaluated after overexpression or knockdown of METTL14 in CNE2 and HONE1 cells by RT-qPCR.

**E.** Correlation analysis of METTL14 and ANKRD22 mRNA in 28 NPC tissues and 7 non-tumor NPE tissues.

**F.** Correlation between METTL14 and ANKRD22 in 80 NPC tissues were analyzed using IHC results. Scale bar: 200 ×, 100 μm. Left panel: representative images of METTL14 and ANKRD22 in NPC tissues and NPE tissues. Right panel: correlation between METTL14 and ANKRD22 expression in NPC tissues and NPE tissues.

**G, H.** Overexpression and knockdown of ANKRD22 were evaluated by RT-qPCR (**G**) and western blotting (**H**).

**I.** Wound healing assays were employed to assess the cell migration ability after overexpression or knockdown of ANKRD22 in CNE2 and HONE1 cells. Representative images (left panel) and statistical analysis (right panel) were presented. Images were acquired at 0 and 24 hours. Scale bar: 200 μm.

**J.** Transwell migration and invasion assays were employed to assess the migration and invasion abilities after overexpression or knockdown of ANKRD22 in CNE2 and HONE1 cells. Representative images (left panel) and statistical analysis (right panel) were presented. Scale bar: 100 μm.

**K, L.** The cell proliferation ability after overexpression or knockdown of ANKRD22 in CNE2 and HONE1 cells were analyzed using MTT (**K**) and colony formation assays (**L**).

Data were presented as mean ± SD. ***p* < 0.01, ****p* < 0.001, *****p* < 0.0001.

**
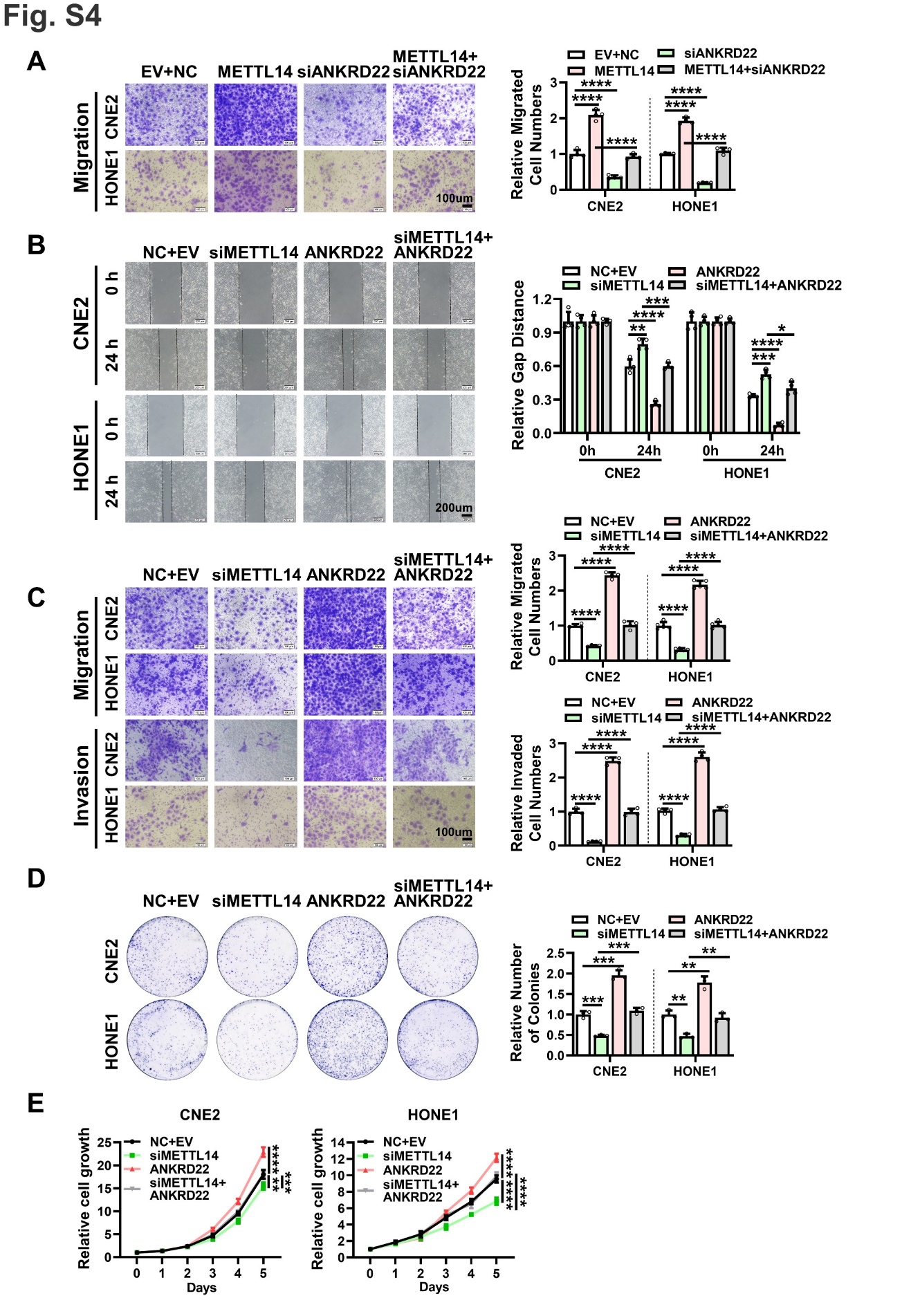
**

**Supplementary Fig. 4 METTL14 upregulates the expression of ANKRD22 to promote NPC malignant progression**

**A.** Transwell migration assays were employed to analyze the cell migration ability after co-transfection of METTL14 overexpression vector and ANKRD22 siRNA in CNE2 and HONE1 cells. Scale bar: 100 μm.

**B, C.** Wound healing assays (**B**) and Transwell assays (**C**) were employed to evaluate the migration and invasion ability after co-transfection of METTL14 siRNA and ANKRD22 overexpression vector in CNE2 and HONE1 cells. D, Scale bar: 200 μm. E, Scale bar: 100 μm.

**D, E.** Colony formation assays (**D**) and MTT assays (**E**) were employed to analyze the cell proliferation ability after co-transfection of METTL14 siRNA and ANKRD22 overexpression vector in CNE2 and HONE1 cells.

Data were presented as mean ± SD. ***p* < 0.01, ****p* < 0.001, *****p* < 0.0001.

**
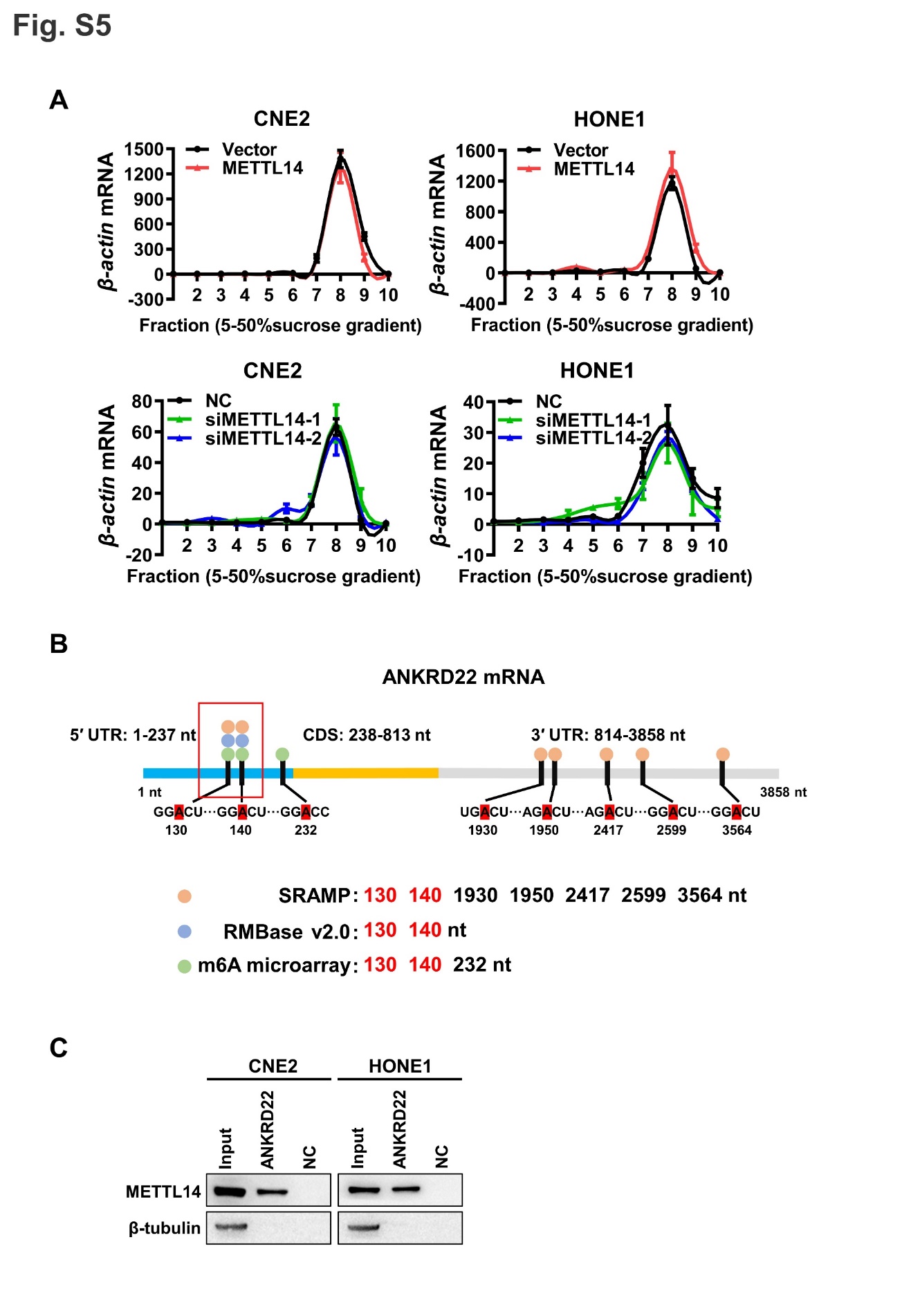
**

**Supplementary Fig. 5 METTL14 enhances the stability and translation of ANKRD22 mRNA in an m^6^A-dependent manner**

**A.** Polysome analysis was employed to assess the translation efficiency of β-actin mRNA after overexpression or knockdown of METTL14 in CNE2 and HONE1 cells. RNAs in different ribosome fractions were extracted and analyzed by RT-qPCR.

**B.** m^6^A modification sites on ANKRD22 as predicted by SRAMP and RMBase v2.0 and shown by m^6^A and gene expression profiling microarray data with high confidence. Yellow circles: locations of SRAMP-predicted m^6^A modification sites. Blue circles: location of m^6^A modification sites predicted by RMBase v2.0. Green circles: locations of m^6^A modification sites indicated by m^6^A and gene expression profiling microarray data. Red boxes: m^6^A modification sites with the highest confidence.

**C.** RNA pull-down assays were performed to assess the binding of ANKRD22 mRNA with METTL14 in CNE2 and HONE1 cells. NC: magnetic beads only.

**
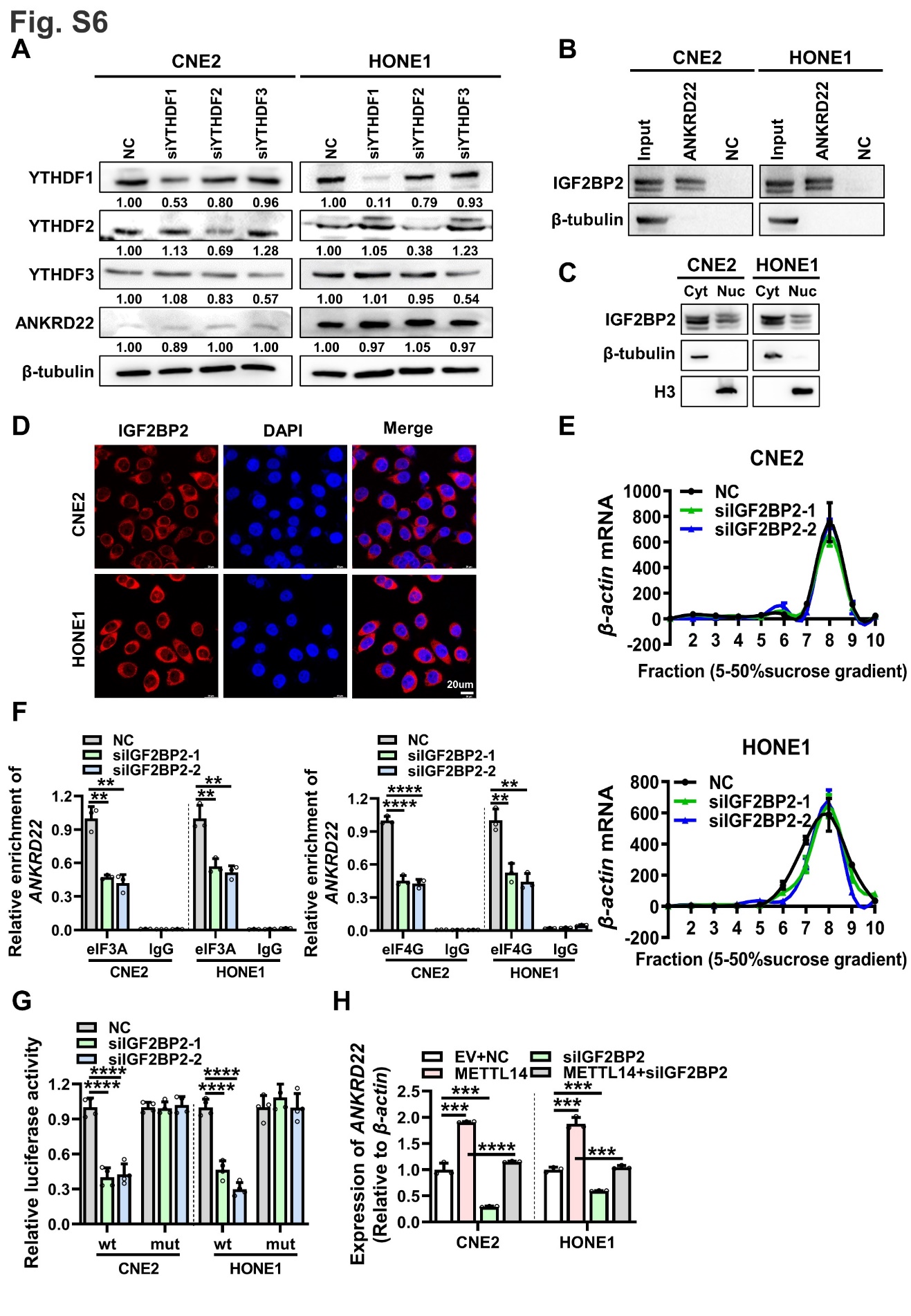
**

**Supplementary Fig. 6 IGF2BP2 recognizes METTL14-mediated m^6^A modification on ANKRD22 mRNA**

**A.** Protein levels of ANKRD22 in CNE2 and HONE1 cells after knockdown of YTHDF1/2/3 were detected by western blotting.

**B.** RNA pull-down assays were conducted to assess ANKRD22 mRNA binding with IGF2BP2 in CNE2 and HONE1 cells. NC: magnetic beads only.

**C.** Protein nuclear-cytoplasmic fractionation assays were employed to assess the ANKRD22 localization in CNE2 and HONE1 cells. H3 was served as a nuclear marker, and β-tubulin was a cytoplasmic marker. Cyt: cytoplasm, Nuc: nucleus.

**D.** IF assays were conducted to determine the IGF2BP2 localization in CNE2 and HONE1 cells. Cell nuclei were counterstained with DAPI (blue). Scale bar: 20 μm.

**E.** Polysome analysis was employed to evaluate the translation efficiency of β-actin mRNA after knockdown of IGF2BP2 in NPC cells. RNAs from different ribosome fractions was extracted and analyzed by RT-qPCR.

**F.** RIP RT-qPCR assays were employed to assess the binding of eIF3A (left panel) or eIF4G (right panel) to ANKRD22 mRNA after knockdown of IGF2BP2 in CNE2 and HONE1 cells.

**G.** Luciferase reporter assays were employed to examine the effect of knocking down METTL14 on the luciferase activity from the ANKRD22 5′ UTR m^6^A sequence in CNE2 and HONE1 cells.

**H.** The effect of co-transfection of METTL14 overexpression vector and IGF2BP2 siRNA on ANKRD22 mRNA level in NPC cells was examined using RT-qPCR.

Data were presented as mean ± SD. ***p* < 0.01, ****p* < 0.001, *****p* < 0.0001.

**
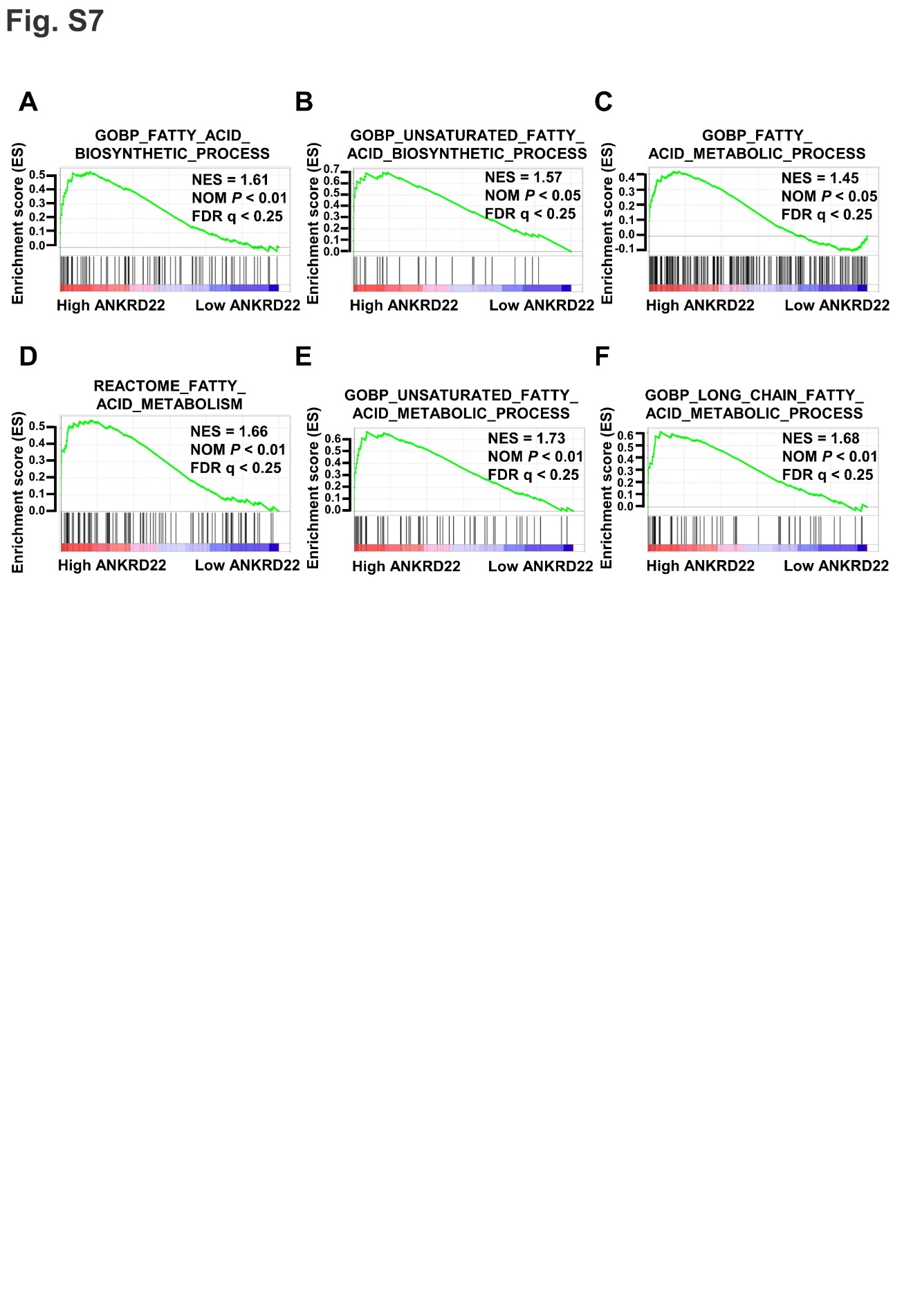
**

**Supplementary Fig. 7 GSEA analysis of GSE12452 data for possible signaling pathways associated with ANKRD22**

**A, B.** Fatty acid biosynthesis.

**C-F.** Fatty acid metabolism.

**
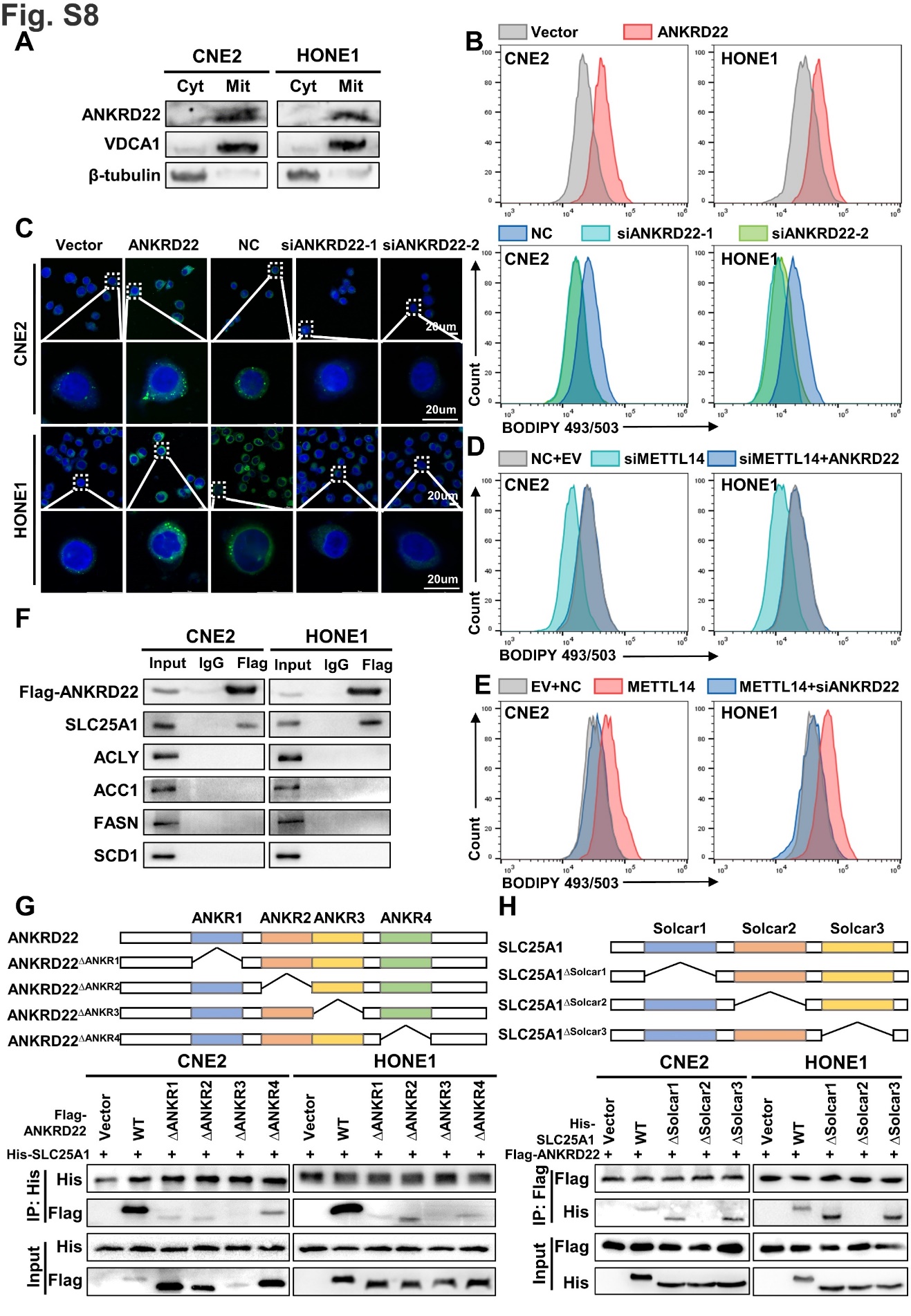
**

**Supplementary Fig. 8 METTL14 prompts lipid metabolism in NPC cells by upregulating ANKRD22 expression**

**A.** Western blotting showing that ANKRD22 was localized in mitochondria. Mitochondria were isolated using a mitochondrial isolation kit. VDAC1 served as a mitochondrial marker, and β-tubulin was a cytoplasmic marker. Cyt: cytoplasm, Mit: mitochondrion.

**B.** Flow cytometry assays were performed to analyze the lipid contents after overexpression or knockdown of ANKRD22 in CNE2 and HONE1 cells. Statistical results are presented in Fig. 6B.

**C.** IF assays were performed to evaluate the lipid contents (green) after overexpression or knockdown of ANKRD22 in CNE2 and HONE1 cells. Cell nuclei were counterstained with DAPI (blue). Scale bar: 20 μm.

**D.** Flow cytometry assays were performed to analyze the lipid contents after co-transfection of METTL14 siRNA and ANKRD22 overexpression vector in CNE2 and HONE1 cells. Statistical results are presented in Fig. 6C.

**E.** Flow cytometry assays were performed to analyze the lipid contents after co-transfection of METTL14 overexpression vector and ANKRD22 siRNA in CNE2 and HONE1 cells. Statistical results are presented in Fig. 6C.

**F.** Co-IP assays were performed to analyze the interaction between ANKRD22 and SLC25A1, ACLY, ACC1, FASN, SCD1 in CNE2 and HONE1 cells using an anti-Flag antibody.

**G:** Co-IP assays showing direct interaction between ANKRD22 and SLC25A1. The schematic diagram indicates the relevant domains and deletion mutants of ANKRD22 protein.

**H:** Co-IP assays showing direct interaction between SLC25A1 and ANKRD22. The schematic diagram indicates the relevant domains and deletion mutants of SLC25A1 protein.

**
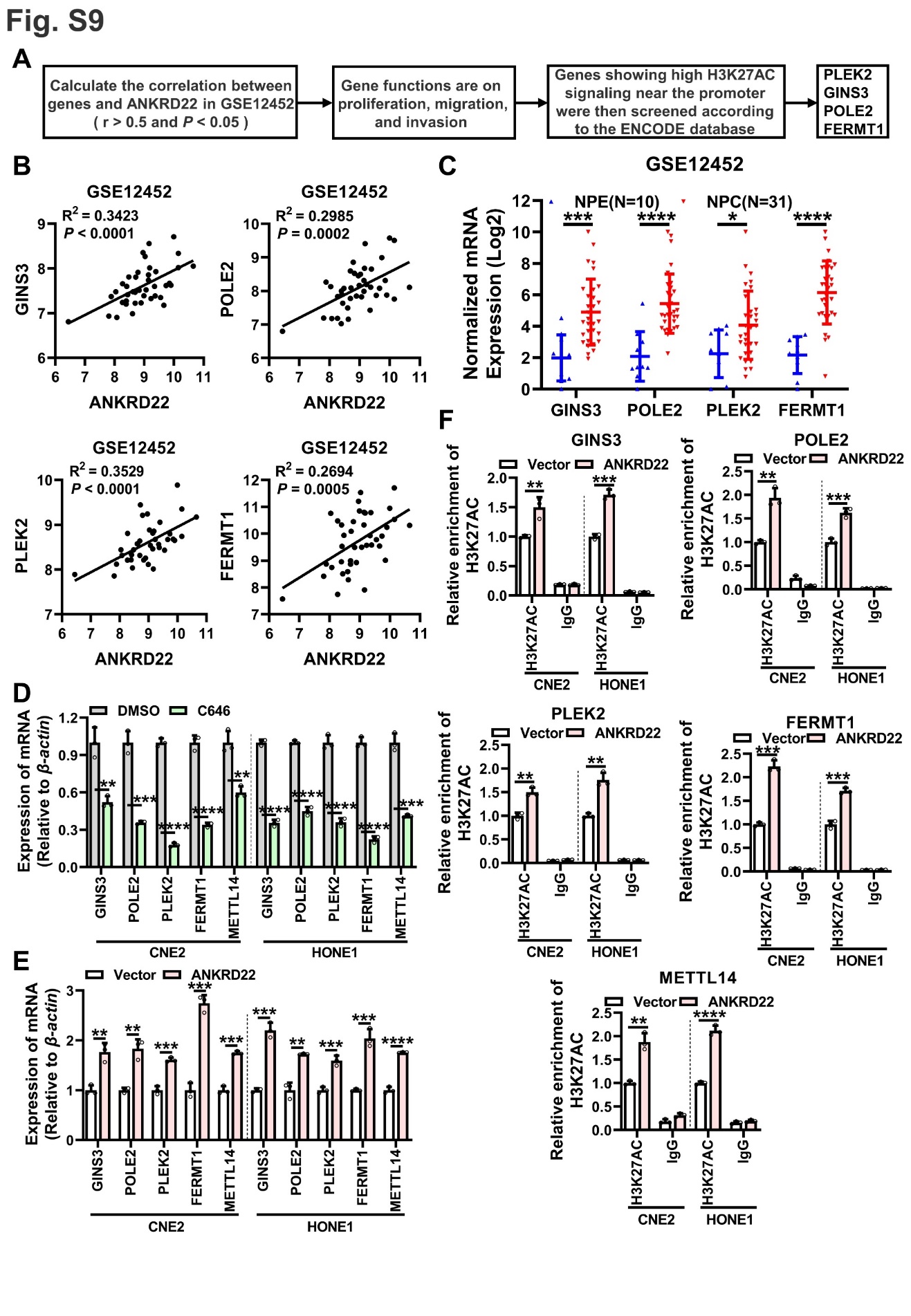
**

**Supplementary Fig. 9 ANKRD22 activates downstream gene transcription through enhancing H3K27AC**

**A.** Flowchart for screening downstream targets of ANKRD22. Molecules with strong correlation (r > 0.5, *p* < 0.05) with ANKRD22 in NPC dataset GSE12452 were identified and the function of these genes was related to proliferation, migration, and invasion, while ChIP-seq data from NPC cells showed high H3K27AC signals near their promoters.

**B.** The correlation of ANKRD22 with the expression of GINS3, POLE2, PLEK2, and FERMT1 genes in NPC dataset GSE12452.

**C.** The expression of GINS3, POLE2, PLEK2, FERMT1 genes in NPC dataset GSE12452.

**D.** The effect of C646 (20 μM) on GINS3, POLE2, PLEK2, FERMT1, and METTL14 mRNA levels in CNE2 and HONE1 cells was assessed using RT-qPCR.

**E.** The effect of overexpression of ANKRD22 on GINS3, POLE2, PLEK2, FERMT1, and METTL14 mRNA levels in CNE2 and HONE1 cells was determined using RT-qPCR.

**F.** ChIP-qPCR assays were employed to analyze H3K27AC levels near the promoters of GINS3, POLE2, PLEK2, FERMT1, and METTL14 after overexpression of ANKRD22 in CNE2 and HONE1 cells.

Data were presented as mean ± SD. **p* < 0.05, ***p* < 0.01, ****p* < 0.001, *****p* < 0.0001.

**
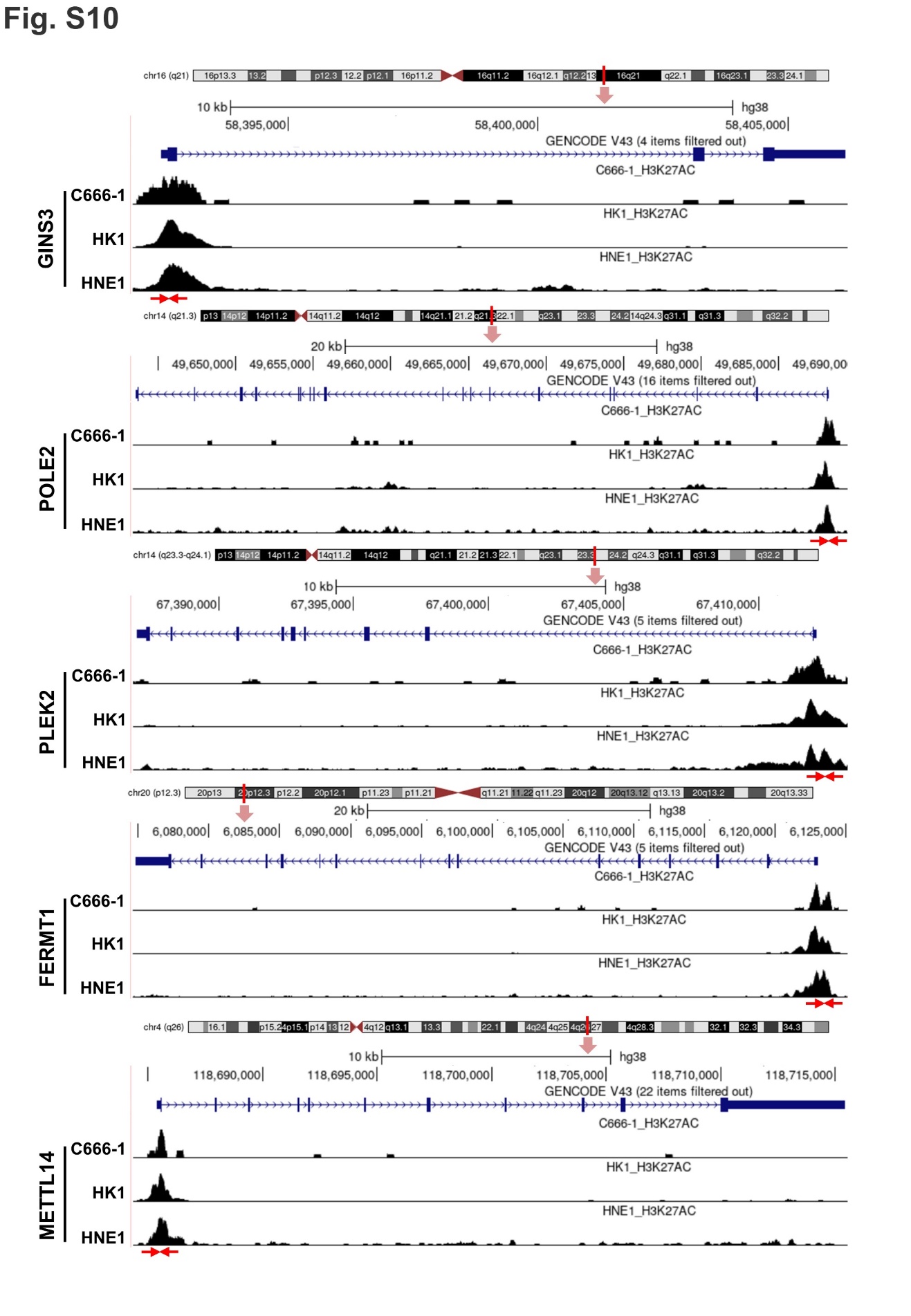
**

**Supplementary Fig. 10 UCSC visualization revealed the presence of H3K27AC near the promoters of GINS3, POLE2, PLEK2, FERMT1, and METTL14 genes in C666-1, HK1, and HNE1 cells.** Red arrows indicate ChIP-qPCR amplified regions.

**
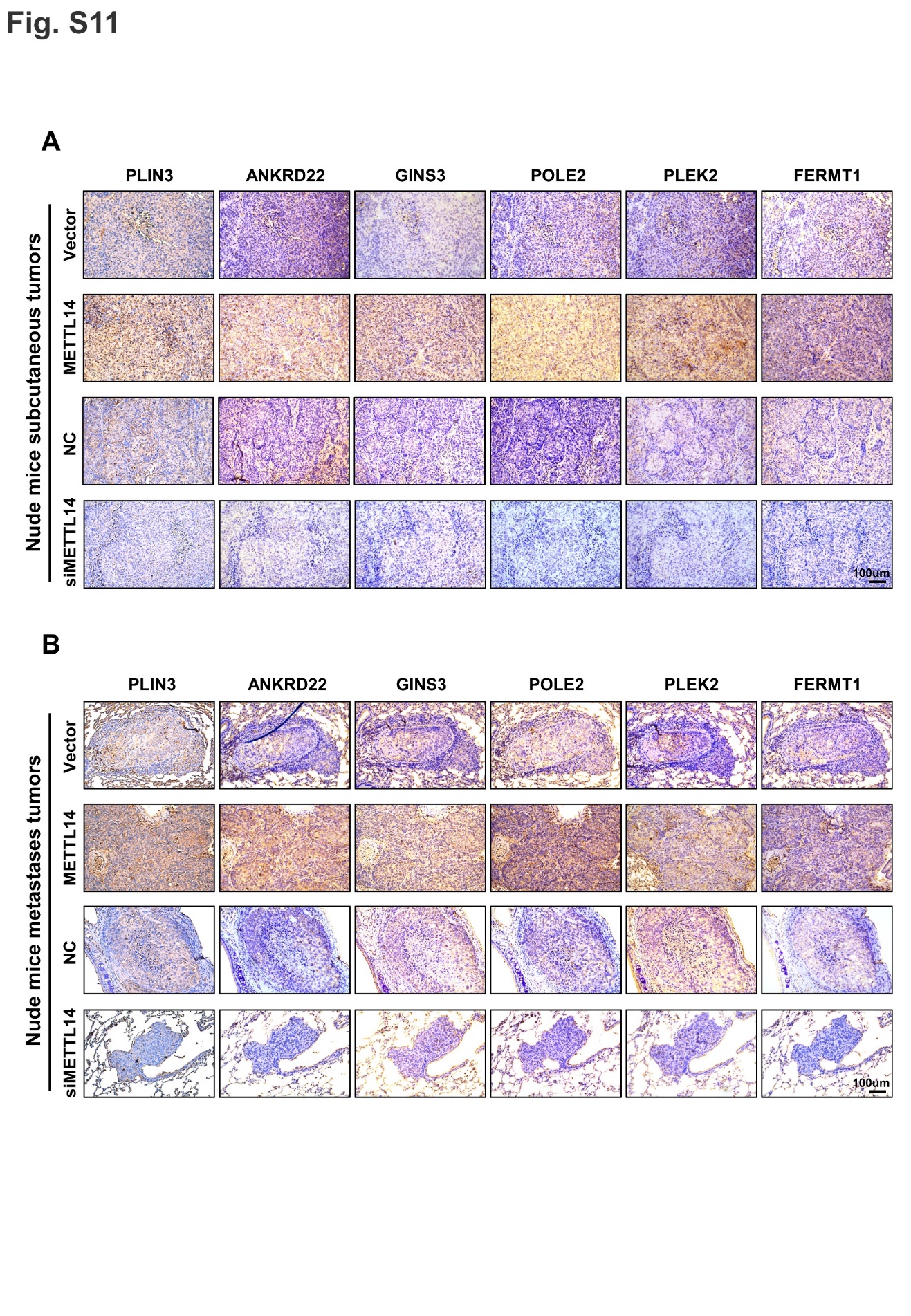
**

**Supplementary Fig. 11 Expression of ANKRD22 and its downstream genes in nude mouse subcutaneous tumor model and lung metastasis model revealed by IHC.**

**A.** Expression of PLIN3, ANKRD22, GINS3, POLE2, PLEK2, and FERMT1 was assessed using IHC in nude mouse subcutaneous tumor model. Scale bar: 200 ×, 100 μm.

**B.** Expression of PLIN3, ANKRD22, GINS3, POLE2, PLEK2, and FERMT1 was assessed in nude mouse lung metastasis model using IHC. Scale bar: 200 ×, 100 μm.

**Supplementary Table 1. Clinicopathological data for 28 NPC and 7 NPE tissues-used for RT-qPCR**

**Supplementary Table 2. Clinicopathological data on 80 paraffin-embedded NPC tissues and the expression of METTL14, ANKRD22 in these samples measured by IHC**

**Supplementary Table 3. Clinicopathological data on 70 paraffin-embedded NPC tissues and the expression of METTL14, ANKRD22, GINS3, POLE2, PLEK2 and FERMT1 in these samples measured by IHC**

**Supplementary Table 4. List of siRNAs**

**Supplementary Table 5. List of RT-qPCR primers**

**Supplementary Table 6. List of primary antibodies for IHC, western blotting, m^6^A dot blot, IF, IP and ChIP**

**Supplementary Table 7. List of 739 genes with both differential m^6^A levels and differential mRNA levels**

**Supplementary Table 8. List of genes in GSE12452 with a correlation coefficient greater than 0.5 with ANKRD22**
